# Single-cell RNA-seq reveals the genesis and heterogeneity of tumor microenvironment in pancreatic undifferentiated carcinoma with osteoclast-like giant-cells

**DOI:** 10.1186/s12943-022-01596-8

**Published:** 2022-06-22

**Authors:** Xinbo Wang, Jiaying Miao, Sizhen Wang, Rongxi Shen, Shuo Zhang, Yurao Tian, Min Li, Daojun Zhu, Anlong Yao, Wei Bao, Qun Zhang, Xingming Tang, Xingyun Wang, Jieshou Li

**Affiliations:** 1 Research Institute of General Surgery, Jinling Hospital, Nanjing University School of Medicine, Nanjing, 210002 Jiangsu, China; 2 International Genome Center, Jiangsu University, Zhenjiang, 212013 Jiangsu China; 3 State Key Laboratory of Pharmaceutical Biotechnology, School of Life Sciences, NJU Advanced Institute for Life Sciences (NAILS), Nanjing University, 163 Xianlin Road, Nanjing, 210046 Jiangsu China; 4 Department of Pathology, Jinling Hospital, Nanjing University School of Medicine, Nanjing, 210002 Jiangsu China; 5 Department of Medical Oncology, Jinling Hospital, Nanjing University School of Medicine, Nanjing, 210002 Jiangsu China; 6 Hongqiao International Institute of Medicine, Tongren Hospital, Shanghai Jiao Tong University School of Medicine, Shanghai, China

**Keywords:** UCOGCP, scRNA-seq, Pancreatic cancer, Tumor microenvironment, PDAC

## Abstract

**Background:**

Undifferentiated carcinoma with osteoclast-like giant cells (OGCs) of pancreas (UCOGCP) is a rare subtype of pancreatic ductal adenocarcinoma (PDAC), which had poorly described histopathological and clinical features.

**Methods:**

In this study, single-cell RNA sequencing (scRNA-seq) was used to profile the distinct tumor microenvironment of UCOGCP using samples obtained from one UCOGCP patient and three PDAC patients. Bioinformatic analysis was carried out and immunohistochemical (IHC) staining was used to support the findings of bioinformatic analysis. After quality control of the raw data, a total of 18,376 cells were obtained from these four samples for subsequent analysis. These cells were divided into ten main cell types following the Seurat analysis pipeline. Among them, the UCOGCP sample displayed distinct distribution patterns from the rest samples in the epithelial cell, myeloid cell, fibroblast, and endothelial cell clusters. Further analysis supported that the OGCs were generated from stem-cell-like mesenchymal epithelial cells (SMECs).

**Results:**

Functional analysis showed that the OGCs cluster was enriched in antigen presentation, immune response, and stem cell differentiation. Gene markers such as LOX, SPERINE1, CD44, and TGFBI were highly expressed in this SMECs cluster which signified poor prognosis. Interestingly, in myeloid cell, fibroblasts, and endothelial cell clusters, UCOGCP contained higher percentage of these cells and unique subclusters, compared with the rest of PDAC samples.

**Conclusions:**

Analysis of cell communication depicted that CD74 plays important roles in the formation of the microenvironment of UCOGCP. Our findings illustrated the genesis and function of OGCs, and the tumor microenvironment (TME) of UCOGCP, providing insights for prognosis and treatment strategy for this rare type of pancreatic cancer.

**Supplementary Information:**

The online version contains supplementary material available at 10.1186/s12943-022-01596-8.

## Introduction

Among pancreatic tumors, undifferentiated carcinoma is a type of rare and highly aggressive subtype, which tends to be histologically characterized as high interstitial, pleomorphic large cell carcinoma, spindle cell carcinoma, and sometimes sarcomatoid carcinoma [1, 2]. Sometimes, osteoclast-like giant cells were observed with undifferentiated carcinoma in pancreatic tumors (UCOGCP), which accounted for less than 1% of the pancreatic adenocarcinoma [3, 4]. Differing from pancreatic tumors without OGCs, UCOGCP tissues tended to be larger in size, sometimes reached 5 to 10 cm in diameter when diagnosed, accompanied with polypoid growth or cystic lesion [5]. In addition, UCOGCP has been frequently noted to coexist with other types of pancreatic ductal adenocarcinoma (PDAC) or mucinous cystic tumors (MCN) [6–8]. The prognosis of UCOGCPs is controversial. Most of the UCOGCPs had poor prognosis compared to other PDACs [9–11], with the median or average survival of less than one year [11, 12]. This might be attributed to the fact that UCOGCP tended to be diagnosed at a late stage and the tumors tend to recur soon after surgery [13, 14] or ineligible for resection [15]. With the improvement of the diagnostic technology, UCOGCPs could be diagnosed more effectively [16], and some studies reported that the prognosis of UCOGCP is significantly better than that of PDAC, especially in patients with “pure” OCGs [17].

The occurrence, growth and metastasis of cancers are closely related to the internal and external environment of tumor cells. The structure, function, and metabolism of tumor cells and the cells in the tumor microenvironment (TME) facilitate tumor cells survival and development [18]. Since UCOGCP is extremely rare, the heterogeneity of its TME is unclear. In “pure” UCOGCP lesion or the UCOGC area of the mixed pancreatic cancer, there are mainly three types of cells: the non-neoplastic OGCs, the neoplastic mononuclear cells, as well as the mononuclear histiocytes (MCHs) [19]. Immunohistochemical straining showed that MCHs expressed CD163, a gene marker for tumor-associated macrophages (TAM2) [20], whereas OGCs and partial mononuclear histiocytic cells express CD68 [10]. Some studies indicated that UCOGCPs are inert [4, 21], especially for pure OGCs in pancreatic tissues [22], and OGC may phagocytize tumoral cells [23]. UCOGCPs are classified into a rare type of PDAC and OGCs was considered epithelial genesis, as they show similar genetic alterations with PDAC, like containing mutation in KRAS [19]. However, based on studies using microscopy, immunohistochemical staining and whole exome sequencing, the genesis of OGCs, whether it is a non-neoplastic mesenchymal, neoplastic mesenchymal or epithelial origin, remains unclear, and their functions remain debating and need more investigation [10, 22, 24]. Due to the rarity of UC-OGC, there is currently no standardized treatment plan, and surgery is the main treatment of choice, which had high risks of recurrence or metastasis [13, 14]. The efficacy of radiotherapy, chemotherapy and immunotherapy remains to be evaluated [25]. Therefore, understanding the histogenesis of OGCs and the heterogeneity of their tumor microenvironment will help us tailoring standardized treatment plan for UGOGCP.

Compared with traditional bulk RNA-seq, the generation and development of single cell RNA-seq (scRNA-seq) provides a solution for describing the heterogeneity of cell clusters and TME in tumor tissues [26]. scRNA-seq can perform parallel transcription and characterization records of thousands of cells, which can better outline the changes of cell expression patterns and cell–cell interactions. The heterogeneity and immune invasion of pancreatic cancer have been effectively described by scRNA-seq [27]. In this study, we collected samples from one UCOGCP patient and three PDAC patients and analyzed the TME through bioinformatic analysis based on single-cell RNA sequencing (ScRNA-seq) meta data. We revealed that OGCS originated form stem-cell-like mesenchymal epithelial cells (SMECs) and could be a new molecular marker for the prognosis of UCOGCP. In addition, we found that the CD74 pathway plays an important role in tumor associated microphages (TAM) enriched TME formation for UCOGCP, providing potential therapeutic targets for the treatment of UCOGCP.

## Materials and methods

### Patient and sample collection

**Fig. 1:**
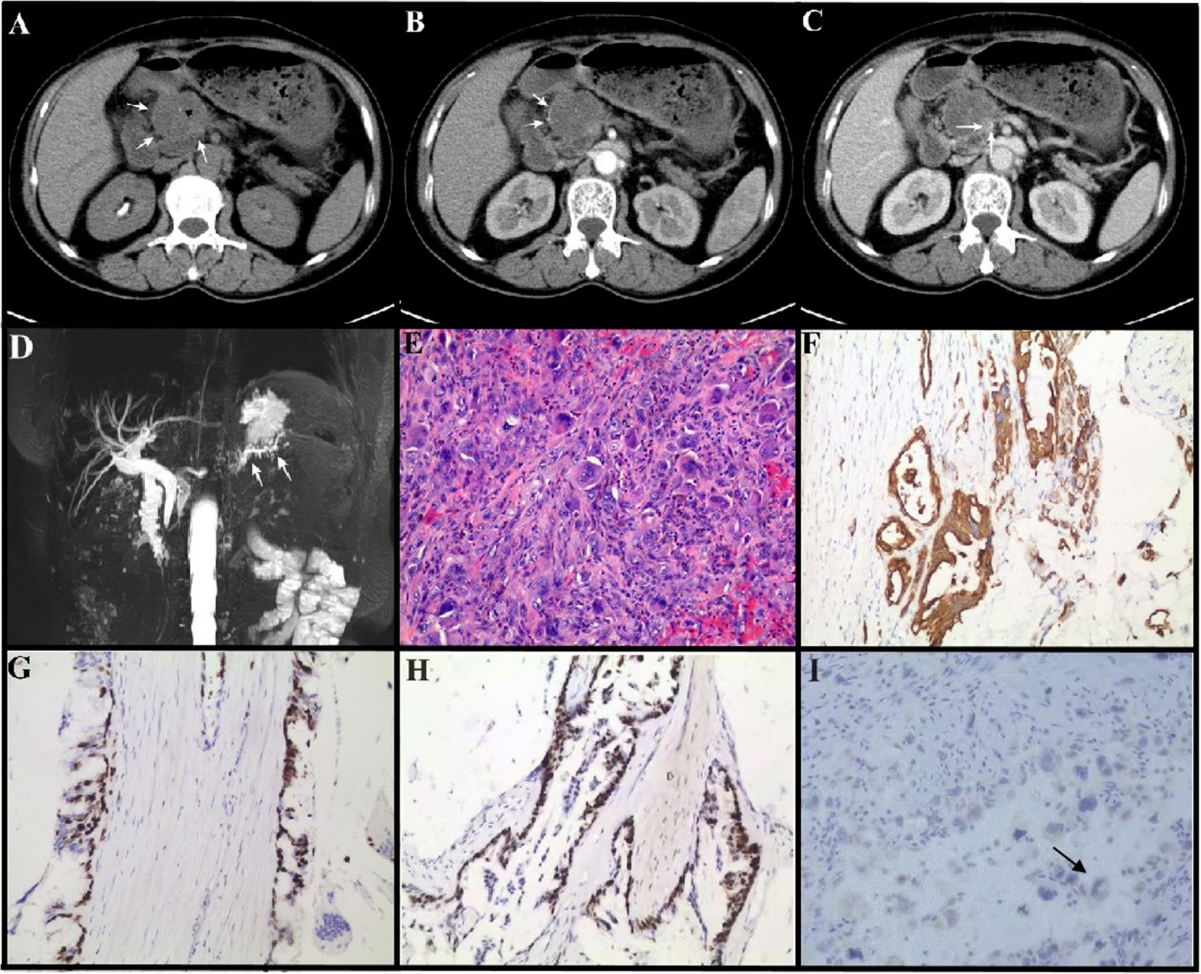
Imaging and pathological analysis of pca_ai1 patient. **A-C**, CT images. Dedicated abdominal CT found an enlarging exophytic 4.5-cm pancreatic head mass (white arrow) with focal calcification (black arrowhead, A). The lesion showed heterogenous low intensity in the edge (arrow, B) relative to pancreatic parenchyma in the pancreatic and portal vein phases with directly protruding into the adjacent superior mesenteric vein (arrow, C). **D**, MRI analysis. A filling defect in the main pancreatic duct (MPD) of the pancreatic head and dilation of the MPD distal to the lesion are evident in MRI, and indicated by arrows in D. **E**, Representative images of H&E staining of surgical removed pancreatic tissue of pca_ai1. Arrows indicated osteoclast-like giant cells (OGCs). **F-I**, Representative images of IHC staining of surgical removed pancreatic tissue of pca_ai1. pan-cytokeratin (CKpan) (**F**), Ki67 (**G**), p53 (**H**), and CD68 (**I**) were used to indicate pleomorphic neoplastic cells and OGCs. Arrows in H indicated osteoclast-like giant cells (OGCs) with CD68 positive staining. Bar = 200 µm

Fresh specimens of PDAC with or without UCOGC were collected during surgical resection. Pathological analysis of the samples was carried out blindly by at least two qualified pathologists. All patients in this study provided written informed consents. The patient’s basic information, CT, MRI imaging, and pathology slides are shown in Fig. 1 and Table S1. This work was approved by the Ethics Committee of Jinling Hospital, Nanjing University School of Medicine (No. 2020NZKY-020–01).

### Tissue dissociation and cell purification

Fresh specimens collected from surgery were transported in MACS Tissue Storage Solution (cat 130–100-008, Miltenyi Biotec, Shanghai, China) on ice. DMEM with 10% serum were used to wash tissues three times. Tissues were then dissociated with Tumor Dissociation Kit, human (cat 130–095-929, Miltenyi Biotec, Shanghai, China). Samples were then sieved through a 70 µm cell strainer and centrifuged at 300 g for 5 min and pelleted cells were suspended in red blood cell lysis solution (cat 130–094-183, Miltenyi Biotec, Shanghai, China) to lyse red blood cells. Dissociated cells were washed with PBS with 0.04% BSA (cat B2064, Sigma-Aldrich,). The cell pellets were re-suspended in PBS containing 0.04% BSA and re-filtered through a 35 μm cell strainer. Dissociated single cells were then stained with AO/PI for viability assessment using Countstar Fluorescence Cell Analyzer (Countstar). The single-cell suspension was further enriched with a MACS dead cell removal kit (cat 130–090-101, Miltenyi Biotec, Shanghai, China). Finally, cell suspension with a concentration of 1000 cells/μl in PBS containing 0.04% BSA were used for scRNA-seq.

### Preparation of single-cell suspensions for library construction and scRNA-sequencing

The single-cell gel bead-in-emulsions (GEMs) were generated from single-cell suspensions using 10 × Genomics Chromium Controller (version 3). According to the manufacturer’s instructions, cDNAs were obtained and amplified from the mRNAs in drops by reverse-transcription reactions. The 10 × libraries were sequenced on the NovaSeq sequencing platform (Illumina, San Diego, CA).

### Pre-processing of scRNA-sequencing data

CellRanger (version 4.0.0) was used to obtain the fastq files of the raw data and annotated with the human genome reference sequence (GRCh38). The gene-barcode matrix was then obtained following the Seurat (version 4.0.4) pipeline in R software (version 4.0.5, R-Foundation, Vienna, Austria). Low-quality cells (minimum expression cells > 3, gene numbers < 200, and mitochondrial genes > 15%) were filtered and the rest of cells were employed for bioinformatic analysis.

### Cell clustering analysis, visualization, and annotation

Cell-clustering and sub-clustering analyses were performed with the FindClusters function of the Seurat package with proper resolutions. For the re-clustering of each type of cell clusters, cells with ribosome gene ratio higher than 35% were filtered. Uniform manifold approximation and projection (UMAP) was used to display identified cell clusters and sub-clusters. The cell clusters were annotated based on highly expressed genes, unique expressed genes, and reported canonical cellular markers.

### Pseudotime trajectory analysis

Trajectory analysis of epithelial cells in UCOCGP was preformed following the Monocle 2 pipeline (version 2.18.0) and Monocle 2 pipeline (version 1.0.0; https://github.com/cole-trapnell-lab/monocle3) in R software (version 4.0.5, R-Foundation, Vienna, Austria) [28] with the following major parameters: mean expression > 0.1and num_cells_expressed >  = 10. BEAM function was used to examine the differential expression genes between branches and graph_test function was used to identified co-expressed gene modules.

### Cell communication analysis

Cell communication estimation was preformed using CellPhoneDB Python module (version 0.22), which contained a database of ligands and receptors interaction [29]. Cell interactions were considered relevant if *p* value of ligand–receptor pairs was less than 0.05.

### Functional enrichment analysis

The R package ClusterProfiler 4.0 [30] was used for GO, KEGG, and GSEA analysis of the differential marker genes among subclusters. GSVA package [31] was used for GSVA analysis of the differential marker genes among subclusters. All gene sets came from the Molecular Signatures Database MSigDB (https://www.gseamsigdb.org/gsea/downloads.jsp) [32].

### Regulatory network profiling by SCENIC analysis

Cellular regulatory network was described by transcription factors (TFs) profiling using pySCENIC (version 0.11.2) [33]. Briefly, UCOGCP Seruat S4 object of a read-count matrix from the epithelial clusters containing the top 2000 highly variant gene signatures was used as the input. The matrix was filtered using default parameters. The established gene regulatory networks were shown in heatmap.

### Immunohistochemical (IHC) staining

According to the manufacturer’s instructions, IHC staining was performed using the PANO 7-plex IHC kit (Panovue, Beijing, China). Samples were sliced into a thickness of 5 μm sections. Primary antibody used were: Ki67 (ab92742, Abcam), CD68 (ab213363, Abcam), and KRT81 (ab55407, Abcam). Hematoxylin (SigmaAldrich) was used to stain the nuclei. Representative images were collected using a Zeiss confocal laser-scanning microscope (LSM880).

### TCGA database analysis

GEPIA (Gene Express Profiling Interactive Analysis) and GEPIA2 were used to examine the expression analysis and survival analysis of pancreatic cancer for the marker genes in our work [34].

### Statistical analysis

The statistical analysis was generated using R software (version 4.0.5) and a *p*-value < 0.05 denoted statistically significant.

## Results

### Pathological diagnosis

The sample of the undifferentiated carcinoma with osteoclast-like pancreatic giant cells (UCOGCP) came from a 59-year-old female patient. Abdominal CT found an enlarging exophytic 4.5-cm pancreatic head mass (white arrow in Fig. 1A) with focal calcification (black arrowhead in Fig. 1A). The lesion showed heterogenous low intensity in the edge (arrow in Fig. 1B) relative to pancreatic parenchyma in the pancreatic and portal vein phases, directly protruding into the adjacent superior mesenteric vein (arrow in Fig. 1C). MRI verified a filling defect in the main pancreatic duct (MPD) of the pancreatic head and a dilation of the distal part of the lesion (arrow in Fig. 1D). Subsequent surgical pathology confirmed moderately differentiated mucinous adenocarcinoma with focal features of UCOGC, where the undifferentiated cancer component accounted for about 5% of the tumor mass (black arrow in Fig. 1E). Immunohistochemical (IHC) staining revealed that the pleomorphic neoplastic cells were positive for pan-cytokeratin (CKpan, Fig. 1F), Ki67 (Fig. 1G), and p53 (Fig. 1 H), whereas the OGCs were positive for CD68 only (Fig. 1I). The other three samples are from PDAC, the clinical data were shown in Table S1. To unveil the characteristics of the UCOGC tumor microenvironment, the origination, and the roles of the OGCs in UCOGCP, we performed single-cell RNA sequencing of these four samples.

### ScRNA-seq cellular contribution

**Fig. 2:**
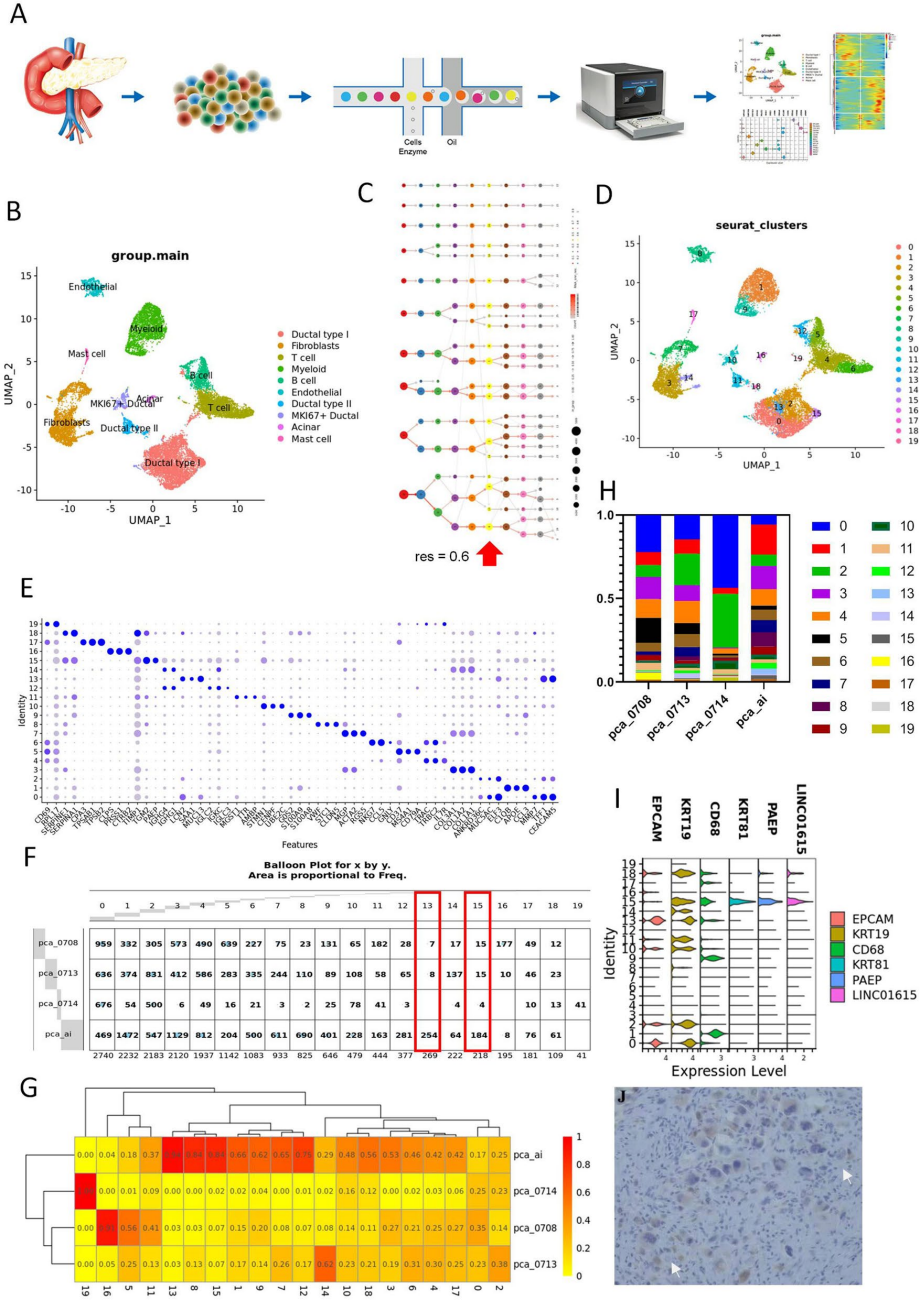
The scRNA-seq summarized the differences in tumor microenvironment between UCOGCP and other PDAC. **A**, Flow chart described the present work. UCOGCP and PDAC samples are dissociated into single cells, captured in 10 × genomic platform for library construction and RNA sequencing. The sequencing results were then undergoing bioinformatics analysis after QC, normalization, PCA. **B**, Uniform manifold approximation and projection (UMAP) showing major clusters learned in Seurat package (4.0.4) in R (4.0.5). D, The proportion of each cluster in different samples. **C**, Clustering tree of total scRNA-seq mate data under different resolutions. Arrows indicated the resolution used in the following figures. **D**, UMAP showing major clusters learned under the resolution of 0.6. **E**, Top three markers of each cluster obtained from “FindAllMarkers” function from Seurat package (4.0.4) were shown in dot plot. **F** and **G**, The distribution of each cluster in each sample shown in balloon plot and heatmap. Clusters containing cells mainly came from UCOGCP sample (pca_ai1) were highlighted in red boxes in F. **H**, The proportion of each cluster in different samples. **I**, Cell markers in cluster 15 shown in violin plot. **J**, Representative images of IHC staining of KRT81 in surgical removed pancreatic tissue of UCOGCP sample (pca_ai1). Representative OGCs with KRT81 positive staining indicated by arrows. Bar = 200 µm

ScRNA-seq meta data were obtained from samples of the patient diagnosed with UCOGC (pca_ai1) and three patients without UCOGC (pca_0708, pca_0713, pca_0714) during tumor resection (Fig. 2A). After initial quality control, 18, 376 cells from all four samples were maintained, and their single-cell transcriptomic data were used for further analysis. Principal component analysis (PCA) displayed the batch effects of the scRNA-seq of four samples (Fig. S1A) and then batch normalization was preformed using Harmony R package (0.1.0) (Fig. S1B). The effects of cell cycle on scRNA-seq meta data of all samples were estimated by the “CellCycleScoring” function in Seurat R package, which showed that little effects of cell cycle exerted on the current data (Fig. S1C). Then the data were clustered with resolution of 0.1 and annotated by marker genes and referring genes from published manuscripts (Fig. 2B; Fig. S1 D-F, Table S2). A total of ten major cell clusters were obtained, that are myeloid cells (LYZ, C1QA, and CD163) [35, 36], endothelial cells (PECAM1 and VWF) [37], NK/T cells (CD3D and NKG7) [37], ductal cells type I, II, and MKI67 (KRT19) [38], B cells (CD79A and IGKC) [39], acinar cells (PRSS1) [38], mast cells (TPSAB1 and TPSB2) [39], and fibroblasts (COL3A1 and COL1A2) [38]. The myeloid cells, endothelial cells, NK/T cells, ductal cells type I, and fibroblasts (COL3A1 and COL1A2) accounted for more than 90% of the total cells (Fig. S1G and H). With the increase of the resolution, these main clusters were divided into more clusters (Fig. 2C, Table S3), and nineteen clusters with distinct gene expression patterns were obtained at the resolution of 0.6 (Fig. 2D and E). The distribution of each sample in different cell types were then profiled (Fig. 2F-H, Fig. S1H). It turned out that pca_0714, which had only 1546 cells for further analysis, mainly clustered into Ductal type I, T cells and myeloid cells, while the rest samples could be found in all subclusters. In addition, UCOGC sample (pca_ai1) showed a different UMAP distribution in comparison with the rest samples in some subclusters. For instance, with the increase of resolution, cluster 13 and cluster 15, that were specific from pca_ai1, could be further isolated from the subcluster of ductal cell type I under the resolution of 0.1. The genetic profiles of the cell clusters also supported that cluster 13 and cluster 15 were from the epithelial lineage cells (Fig. S1I). Furthermore, since cells used in the pca_ai1 sample accounted for about 45% in total cells, a percentage higher than 45% were considered as a concentration in certain subcluster, which are the endothelial cell, the fibroblast cell, and the myeloid cell subclusters, indicating that UCOGC sample (pca_ai1) harboring distinct tumor microenvironment compared with the rest PDAC samples. Therefore, the examination of the tumor microenvironments might reveal the pathological features of UCOGC, such as easy bleeding, necrosis, and bone-like tissue [16]. Moreover, since OGCs might originated from mesenchymal epithelial cells, we then evaluated the expression level of epithelial cell markers EPCAM and KRT19 and the OGC marker CD68 in the two ductal type I clusters of the UCOGC sample (pca_ai1), cluster 13 and 15. It showed that cluster 15 expressed KRT19 but not EPCAM. Moreover, KRT81, PAEP, and LINC01615 were expressed in cluster 15 (Fig. 2I, Table S3). IHC staining of KRT81 were then executed to determine whether cluster 15 contains OGCs and showed that OGCs were KRT81 positive, suggesting that OGCs were most likely originated from mesenchymal epithelial cells rather than myeloid cells (Fig. 2J).

### Heterogeneity of ductal cells type I in UCOGCP

**Fig. 3:**
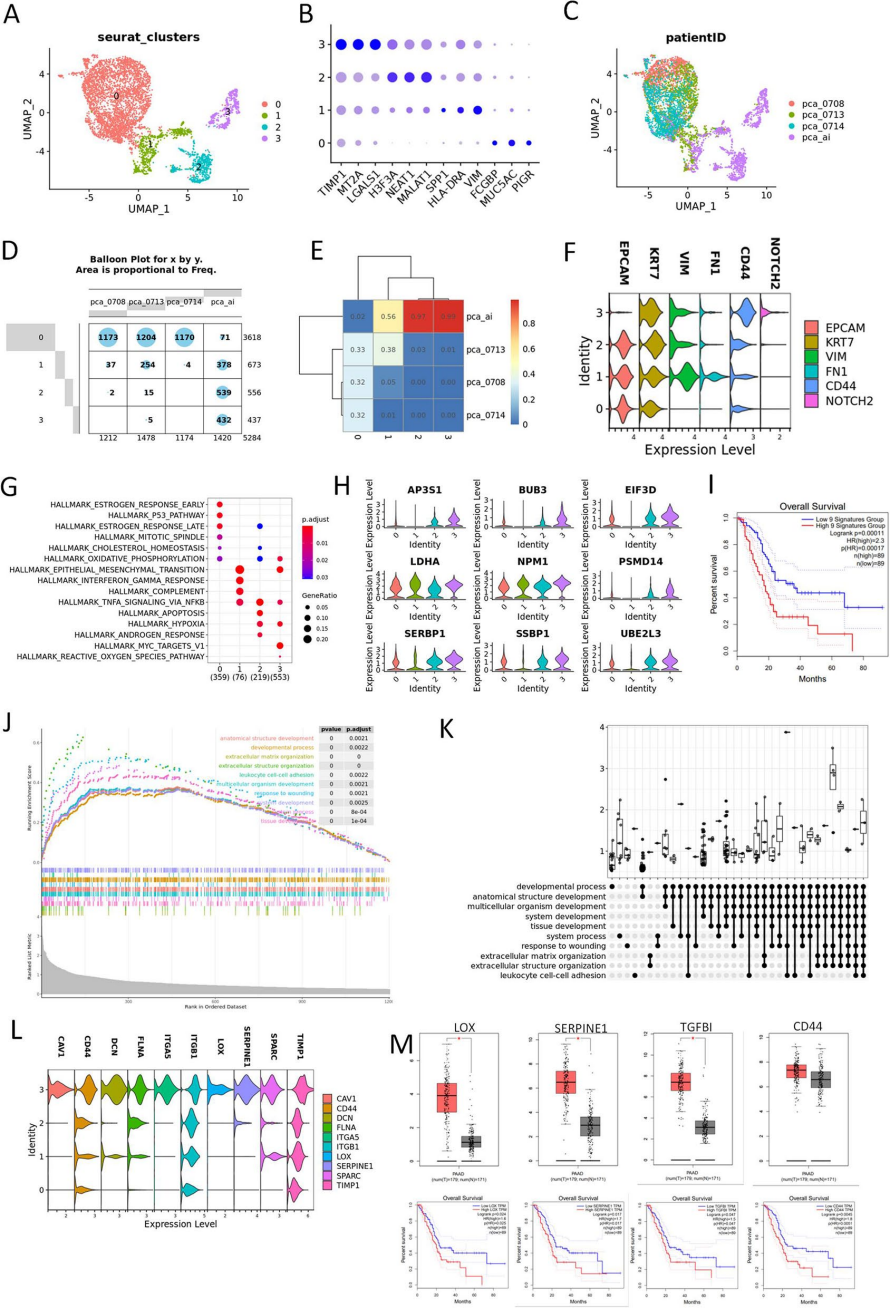
UCOGCP held distinct ductal profile. **A**, Major clusters of the ductal type I cells shown in UMAP. **B**, Top three markers of each cluster obtained from “FindAllMarkers” function from Seurat package (4.0.4) shown in dot plot. **C-E**, The distribution of each cluster in each sample shown in UMAP, balloon plot and heatmap, respectively. **F**, Violin plot showing markers of epithelial cells, cancer stem cells, and epithelial-mesenchymal transition (EMT) cells. **G**, Hallmarks among clusters shown in dot plot. **H**, Expression levels of enriched genes in HALLMAKS-MYC-TARGET-V1. **I**, Survival analysis of gene signatures in H in pancreatic cancer using TCGA-PAAD on website Gepia2 (http://gepia2.cancer-pku.cn/#index). **J** and **K**, GSEGO analysis of gene markers in cluster 2. **L**, Expression level of gene signatures enriched in at least eight of the top GSEGO clusters of cluster 3. **M**, Survival analysis of gene signatures in L in pancreatic cancer using TCGA-PAAD on website Gepia2 (http://gepia2.cancer-pku.cn/#index). N, Expression level and survival analysis of representative genes in L in pancreatic cancer using TCGA-PAAD on website Gepia (http://gepia.cancer-pku.cn/index.html)

To further examine the heterogeneity of ductal cells in UCOGCP, cluster named “ductal type I” in Fig. 2B was then isolated for further analysis. A total of four subclusters were identified with the resolution of 0.1 using “FindClusters” function in Seurat R package (Fig. 3A and B, Table S4). As predicted, UCOGCP (pca_ai1) sample harbored unique epithelial subclusters compared with the rest, subcluster 2 and 3, compared with the other three samples (Fig. 3C-E). Subcluster 3 expressed limited epithelial marker of EPCAM but cancer stem cell marker CD44 and NOTCH2 (Fig. 3F). Hallmark enrichment showed that subcluster 3 were specially enriched in MYC-TARGET_V1 pathway (Fig. 3G, Table S5), and representative gene clusters such as AP3S1, BUB3, EIFD3, LDHA, NMP1, PSMD14, SERBP1, SSBP1, and UBE2L3 affected overall survival based on the TCGA-PAAD data (Fig. 3H and I, Table S5). Furthermore, GSEA analysis of GO BP pathway were calculated for subcluster 3, showing an enrichment in "extracellular matrix organization", "response to wounding", "tissue development", "multicellular organism development", "leukocyte cell–cell adhesion", "anatomical structure development", and "platelet degranulation" pathways (Fig. 3J, Table S6). Ten key genes involved in at least eight of the top 10 pathways were then isolated (Fig. 3K and L, Table S6). The expression level and the survival analysis of these genes were determined in TCGA-PAAD and GTEx-pancreas dataset on GEPIA website. It was found that the high expression levels of most of these genes, especially the LOX, SERPINE1, TGFBI, and CD44, were associated with a poor survival probability (Fig. 3M). When we knocked-down these marker genes on pancreatic cancerous cells in vitro (Fig. S2A-C), compared with the sh-NC group, the viability, migration, and invasion of the pancreatic cancerous cells were decreased significantly, whereas the apoptosis rates were increased significantly, indicating aggressive roles of the LOX, SERPINE1, TGFBI, and CD44 in pcancreatic cancerous cells (Fig. S2D-Q), supporting our informatical analysis.

**Fig. 4:**
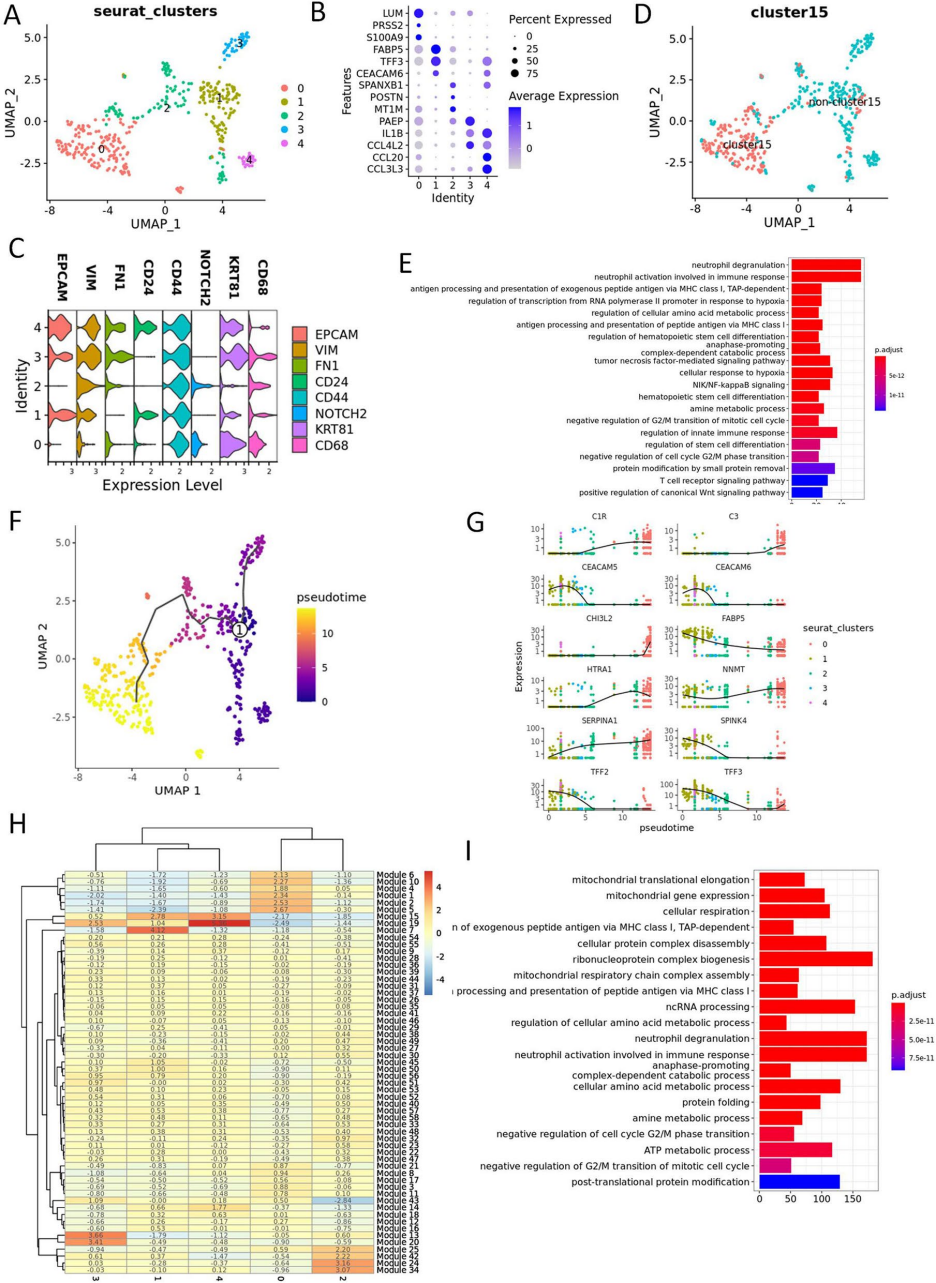
Trajectory analysis and function enrichment of the UCOGCP-specific EMT cells. **A**, Major clusters of the UCOGCP-specific EMT cells shown in UMAP. **B**, Top three markers of each cluster obtained from “FindAllMarkers” function from Seurat package (4.0.4) shown in dot plot. **C**, Expression pattern of interested gene markers. **D**, Mapping of cluster 15 in Fig. 2D. **E**, GO BP enrichment of cluster 0. **F**, Trajectory analysis by monocle 3. **G**, Top ten genes differentially expressed in different clusters. **H**, Heatmap showing gene modules co-expressed in different clusters. **I**, GO BP enrichment of gene modules closely related to cluster 0. Genes in module 1, 2, 5, 6, and 10 were used for GO BP enrichment

The subcluster 3 were then extracted for re-clustering analysis. With the resolution of 0.1, five clusters were obtained (Fig. 4A and B, Table S7). In addition, cluster 4 and 1 expressed cancer stem cell-like cells (CSCLCs) gene markers CD24, CD44, and EPCAM [40, 41], whereas cluster 0 and 2 expressed CD44 and NOTCH2, a gene marker denoted the activation of CSCLCs [42] (Fig. 4C). Cluster 0 was in high overlapping ratio with cluster 15 in Fig. 2D, which seemed to be the cell cluster of our most interests, the OGC cluster. GO biological pathway enrichment analysis was performed on cluster 0 and it was found that this cluster was enriched in antigen presentation, hematopoietic stem cell differentiation and negative regulation of G2/M (Fig. 2E, Table S8). To further examine the changes of gene expression pattern of this cell cluster, trajectory analysis was carried out in monocle 3 R package (Fig. 4F and G). Genes in modules 1, 2, 5, 6, and 10 held close relationship with cluster 0, whose GO biological pathway was also enriched in MHC I antigen presentation, and negative regulation of G2/M, suggesting that this cluster might be multinuclear cells resulted from the dysregulation of cell differentiation from CSCLCs (Fig. 4H and I, Table S9).

### The trajectory analysis of the ductal cells in UCOGCP

**Fig. 5:**
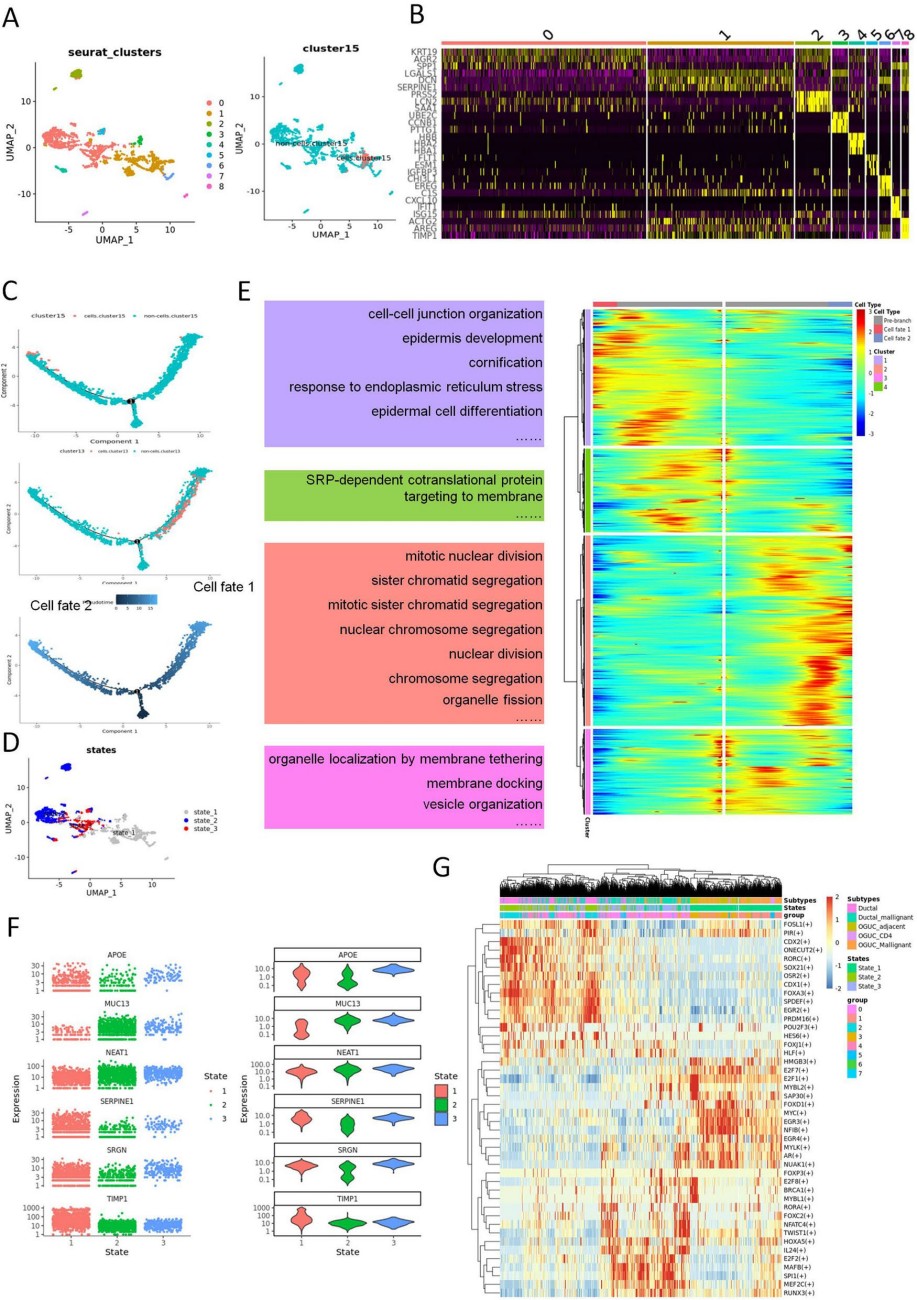
Heterogeneity of epithelial cells in UCOGCP sample (pca_ai1). **A**, UMAP showed the clusters learned by Seurat in R, and the mapping of cluster 15 in Fig. 1D. **B**, Top three markers of each cluster obtained from “FindAllMarkers” function from Seurat package (4.0.4) shown in heatmap. **C**, Trajectory analysis of the epithelial cells in UCOGCP sample. The mapping of the cluster 13/cluster 15 in Fig. 1D and the pseudotime shown in DDRTree reduction in monocle2 package (2.18.0) in R (4.0.5). **D**, Mapping of different states in Seurat clusters shown in UMAP. **E**, The differential expression genes (DEGs) of different branches (different cell fates in C) shown in heatmap. The top GO BP pathways of different clusters in heatmap were listed nearby. **F**, Top five DE genes determined cell fate in C shown among different states. **G**, Transcription factor profile of different states shown in heatmap. Transcription factor profile were estimated by pySCENIC (0.11.2)

To further dissect the developmental progress of all epithelial clusters in UCOGCP, trajectory analysis for epithelial cell clusters were carried out using monocle 2 package in R. Under the resolution of 0.1, a total of ten clusters could be learned in UCOGCP using Seurat analysis pipeline (Fig. S3A and B) and cell cycle held limited effect on the clustering (Fig. S3C). Based on the BaronPancreasData dataset using SingleR R package and representative cell markers from reports, the ten clusters were annotated (Fig. S3 B, E, and F). Mapping of the cluster 15 in Fig. 2D showed that cluster 15 belonged to cluster 4 in UCOGCP (Fig. S3G-I). The ductal clusters in UCOGCP (pca_ai1), cluster 3 and 4 were then isolated for further analysis. After clustering using Seurat pipeline, a total of 8 clusters were obtained (Fig. 5A and B, Table S10). The mapping of cluster 15 in Fig. 2D and clusters in Fig. 2B and Fig. 3A were shown (Fig. 3B, Fig. S4). Trajectory analysis obtained three branches, with the cluster 13 and cluster 15 lied at different branches (Fig. 5C and D; Fig. S5A). Mapping analysis showed that state 1 mainly belong to epithelial clusters that lack of EPCAM (Fig. S5B-E). The expression pattern that determined these two distinct branches were then estimated and separated into four clusters. Genes highly expressed in pre-branch (state 3 in Fig S5A) enriched mainly in “organelle localization by membrane tethering”, “membrane docking”, and “vesicle organization” GO BP pathways, genes enriched in “nuclear division” related pathways were highly expressed in cell fate 2, and genes enriched in “SRP-dependent co-translational protein”, “cell–cell junction organization”, “epidermis development”, and “cornification” were highly expressed in cell fate 1 (Fig. 5E, Tables S11, and S12). Six representative genes determining cell fates were APOE, MUC13, NEAT1, SERPINE1, SRGN, and TIM1 (Fig. 5F). The transcriptional profile of the UCOGCP epithelial cells were then estimated by SCENIC, depicting that transcriptional factor (Tfs), such as E2F1, MYC, EGR4 were highly expressed in the UCOGCP epithelial cluster that lack of EPCAM (Fig. 5G).

### Heterogeneity of the tumor associated myeloid (TAM) cells in UCOGCP

**Fig. 6:**
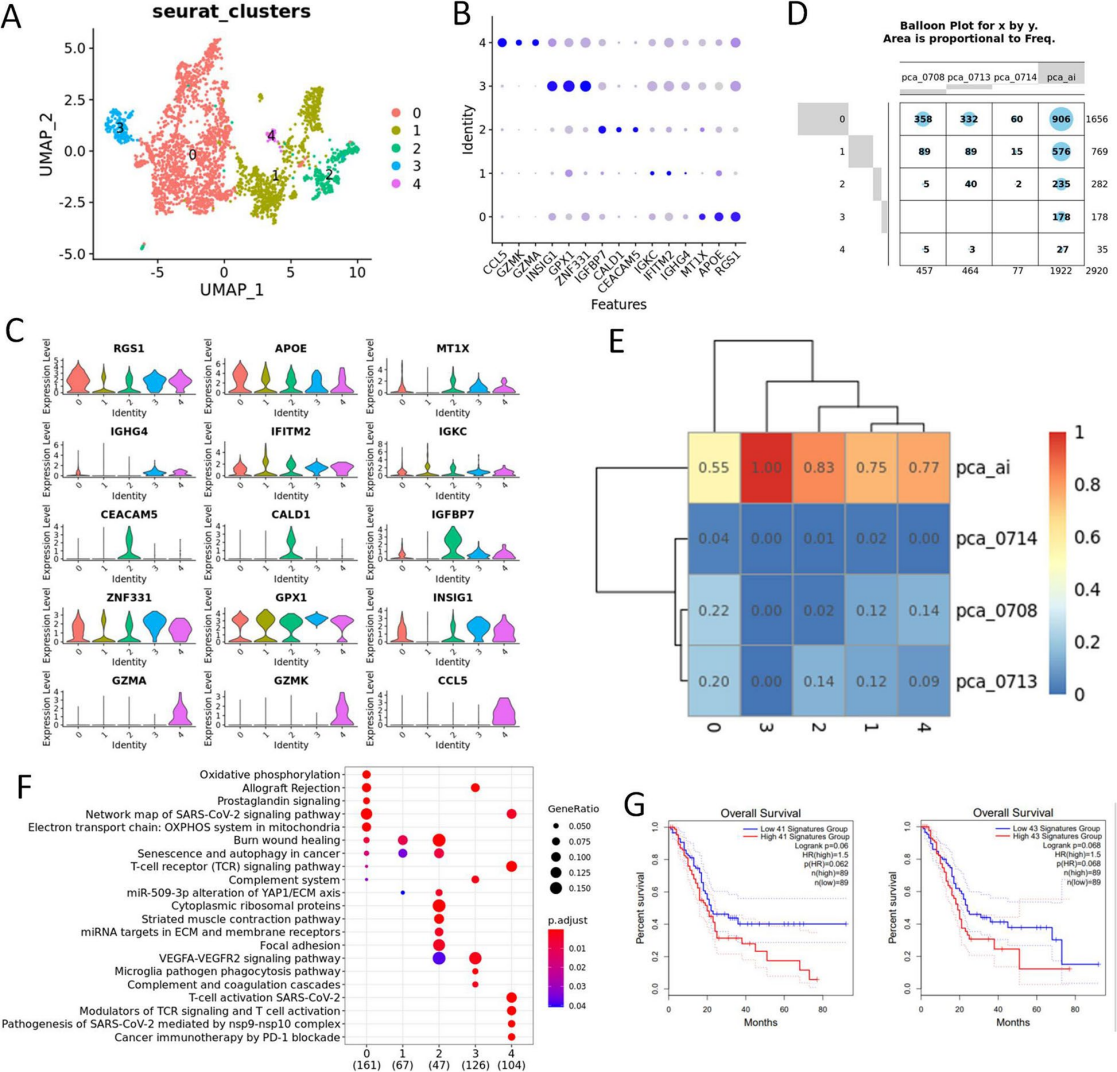
Heterogeneity of tumor associated Myeloid cells. **A**, Major clusters of the myeloid cells of all samples shown in UMAP. **B**, Top three markers of each cluster obtained from “FindAllMarkers” function from Seurat package (4.0.4) shown in dot plot. **C** and **D**, The distribution of each cluster in each sample shown in UMAP, balloon plot and heatmap, respectively. **E**, Violin plot showing the expression level of markers in each cluster. **F**, GO BP enrichment among clusters shown in dot plot. **G**, Survival analysis of gene signatures in cluster 2 and 3 in pancreatic cancer using TCGA-PAAD on website Gepia2 (http://gepia2.cancer-pku.cn/#index)

We then investigated the characteristics of the tumor associated microenvironment in UCOGCP. According to Fig. 2 F and G, we found a large amount of tumor associated myeloid cells, endothelial cells and fibroblasts in UCOGCP (pca_ai1). Therefore, we isolated these clusters for further investigation. In our work, five clusters identified in the TAM cells (Fig. 6A-C) and myeloid cells from UCOGCP (pca_ai1) took dominated percentage in cluster 2 and 3 (Fig. 6D and E). Wikipathway enrichment analysis showed the distinct metabolism pathways of these clusters, showing that cluster 0 was M2 like macrophage cells, cluster 1 was mesenchymal myeloid cells that regulate cancer cell senescence and autophagy, cluster 2 was related to the macrophage mesenchyme transition, cluster 3 was involved in angiogenesis and complement system, and cluster 4 was M1 like macrophage (Fig. 6F, Table S14). Gene signatures of cluster 2 and cluster 3 (logFC > 0.5 and q-value < 0.05) was found to affect the overall survival rate in the TCGA-PAAD dataset (Fig. 6G).

### Heterogeneity of the tumor associated fibroblasts and endothelial cells in UCOGCP

**Fig. 7:**
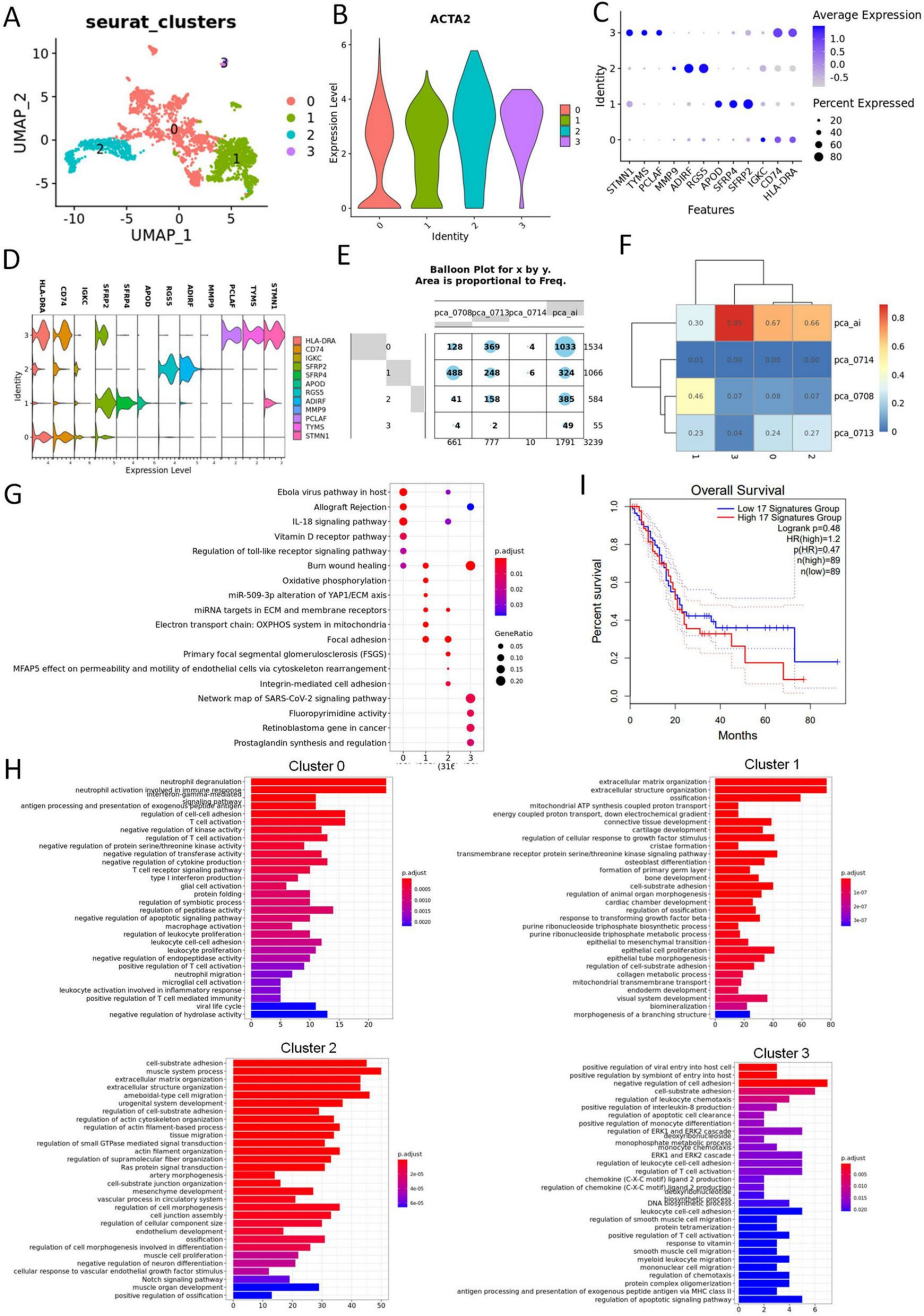
Heterogeneity of tumor associated fibroblast cells. **A**, Major clusters of the tumor associated fibroblast cells of all samples shown in UMAP. **B**, Violin plot showing the expression level of ACTA2 in different clusters. **C**, Top three markers of each cluster obtained from “FindAllMarkers” function from Seurat package (4.0.4) shown in dot plot. **D**, The expression levels of marker genes in different clusters. **E** and **F**, The distribution of each cluster in each sample shown in balloon plot and heatmap, respectively. E and F, Violin plot showing the expression level of markers in each cluster. **G**, Wikipathway enrichment among clusters shown in dot plot. **H**, GO BP enrichment among clusters shown in bar plot. **I**, Survival analysis of gene signatures in cluster 2 and 3 in pancreatic cancer using TCGA-PAAD on website Gepia2 (http://gepia2.cancer-pku.cn/#index)

We then dissected the fibroblasts clusters of all samples, and obtained four major clusters, and all of them expressed the fibroblast marker ACAT2 (Fig. 7A-D, Table S15). We found that cluster three came from the UCOGCP (pca_ai1) (Fig. 7E and F). Wikipathway and GO BP enrichment analysis assisted in unveiling the function of each cluster. Cluster 0 contributed to lymphocyte regulation, cluster 1 was cCAFs (classical CAFs), involving in extracellular matrix related components and cluster 2 was tumor associated PSCs (pancreatic stellate cells), expressing marker genes such as RGS5 and ADIRF (Fig. 7D). In contrast, cluster 3, which was uniquely belonged to UCOGCP (pca_ai1) was associated with leucocyte adhesion and MHCII antigen presentation with special gene markers, such as STMN1, TYMS, and PCLAF (Fig. 7H and D, Table S16).

**Fig. 8:**
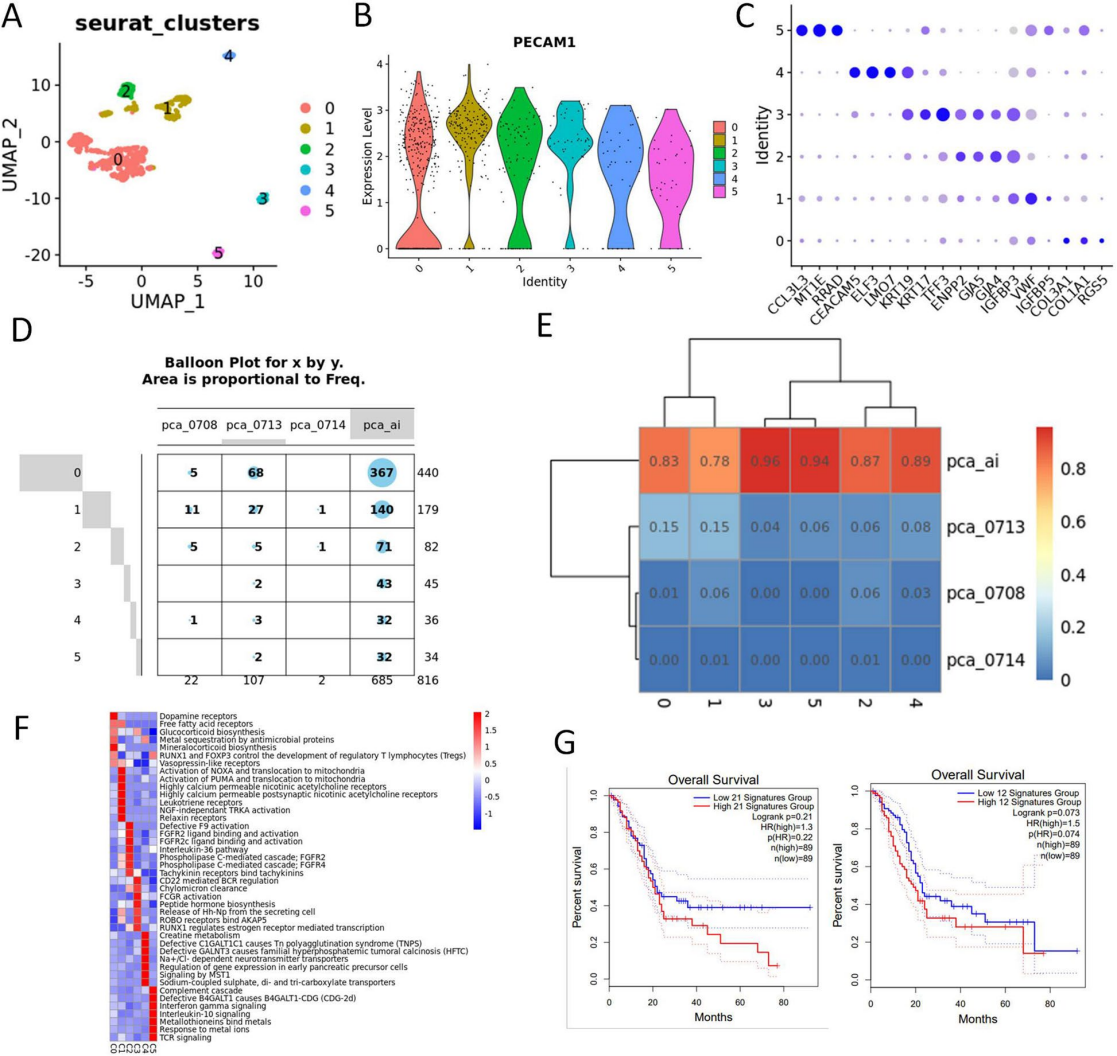
Heterogeneity of tumor associated endothelial cells. **A**, Major clusters of the tumor associated fibroblast cells of all samples shown in UMAP. **B**, Violin plot showing the expression level of PECAM1 in different clusters. **C**, Top three markers of each cluster obtained from “FindAllMarkers” function from Seurat package (4.0.4) shown in dot plot. **D**, The expression levels of marker genes in different clusters. **E** and **F**, The distribution of each cluster in each sample shown in balloon plot and heatmap, respectively. **E**, Violin plot showing the expression level of markers in each cluster. **F**, Wikipathway enrichment among clusters shown in dot plot. **G**, GSVA analysis of KEGG among clusters. **H**, Survival analysis of gene signatures in cluster 3 and 5 in pancreatic cancer using TCGA-PAAD on website Gepia2 (http://gepia2.cancer-pku.cn/#index)

As for tumor associated endothelial cells, that highly expressed marker gene PECAM1, we obtained in six clusters after machine learning (Fig. 8A-C, Table S17). 86% of the endothelial cells came from UCOGCP (pca_ai1), which was consistent with the fact that UCOGCP tumors were often easy bleeding tumors (Fig. 8D and E). Cluster 3 and cluster 5 were dominantly belonged to UCOGCP (pca_ai1). GSVA analysis showed the distinct KEGG pathway enriched for each cluster, where cluster 3 and cluster 5 were both enriched in immunological pathway, such as “CD22 mediated BCR regulation pathway” for cluster 3 and IL-10 signaling for cluster 5 (Fig. 8F). The effects of gene markers (logFC > 1 and q-value < 0.05) in cluster 3 and cluster 5 were also analyzed in the TCGA-PAAD dataset.

### Cell communications in UCOGCP (pca_ai1)

**Fig. 9:**
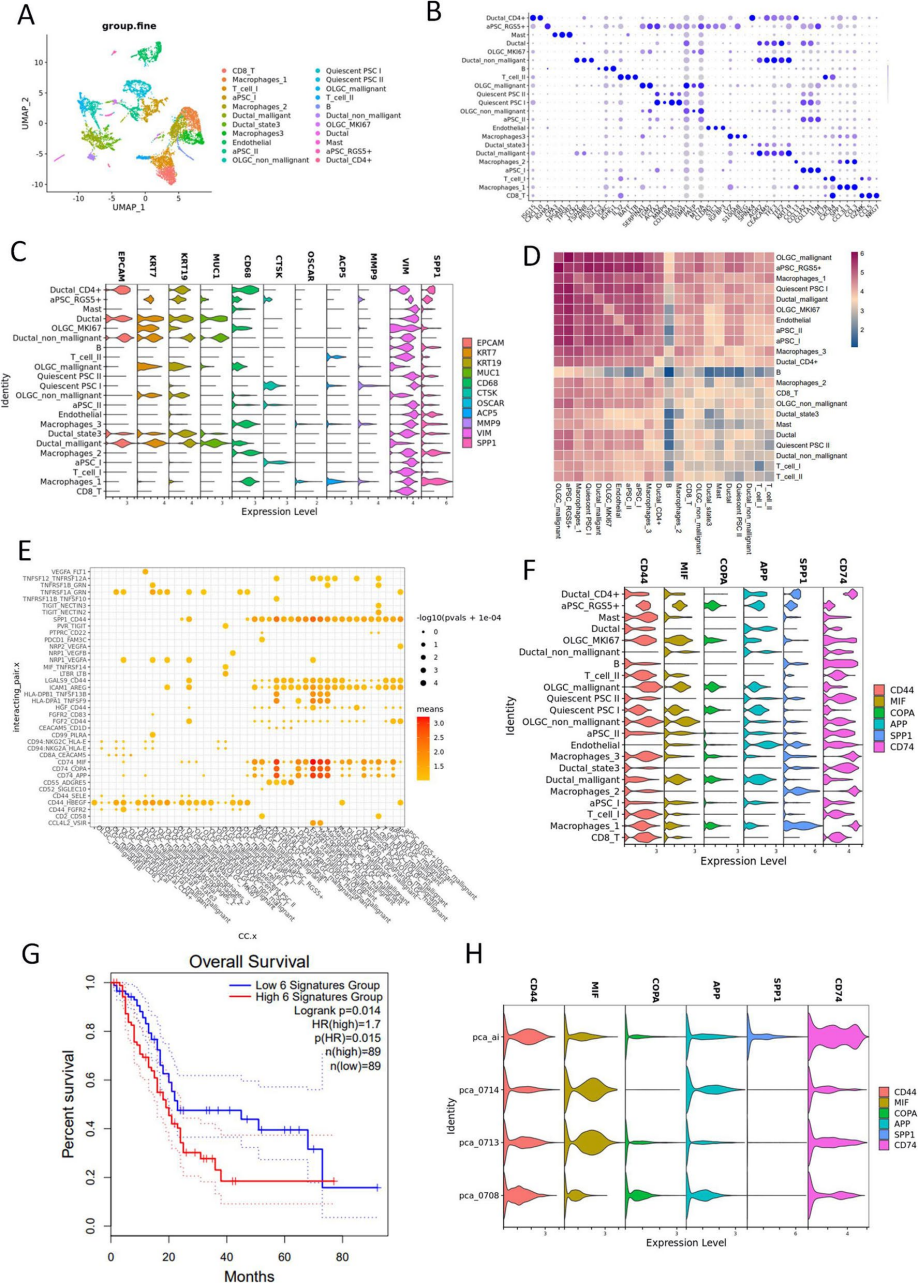
Cell communication analysis in UCOGCP sample (pca_ai1). **A**, Major clusters of the UCOGCP sample shown in UMAP. **B**, Top three markers of each cluster obtained from “FindAllMarkers” function from Seurat package (4.0.4) shown in dot plot. **C**, The expression levels of epithelial marker genes and osteoclasts marker genes in different clusters. **D**, The number of potential ligand–receptor pairs analyzed by pyCellphoneDB (0.24). **E**, Ligand–receptor pairs shown in a bubble plot. **F**, The expression level of ligand–receptor pairs with high means. **G**, Survival analysis of gene signatures in F in pancreatic cancer using TCGA-PAAD on website Gepia2 (http://gepia2.cancer-pku.cn/#index). **H**, The expression level of ligand–receptor pairs among different samples

ScRNA-seq meta data from UCOGCP (pca_ai1) were then used to examine the cell communication of different cell clusters. Based on the above analysis of cell clustering and functional annotation, 22 clusters were annotated in UCOGCP (pca_ai1), which are pca_ai1 CD8_T, Macrophages_1/2/3, T_cell_I/II, aPSC_I, Ductal_malligant, Ductal_state3, Endothelial, aPSC_I/II, OLGC_non_mallignant, Quiescent PSC I/II, OLGC_mallignant, B cell, Ductal_non_mallignant, OLGC_MKI67, Ductal, Mast, aPSC_RGS5 + , and Ductal_CD4 + (Fig. 9A and B, Table S18). To verified that OGCs were osteoclast-like multinucleated cells with no osteoclasts function, we analyzed the gene expression level of osteoclasts markers, such as CTSK, ACP5, OSCAR, and MMP9 [43]. It was found that CTSK, ACP5, and MMP9 was expressed in quiescent PSC_I cluster, which did not express CD68, a well-known IHC marker for OGCs, indicating that the OGCs might only resemble osteoclasts morphologically (Fig. 9C). Cell communication among clusters were then estimated using cellphoneDB. The findings showed strong receptor-ligand interactions between OGCs (OLGC_mallignant cluster) and PSCs, macrophage_I/III, certain epithelial cells, and endothelial cells (Fig. 9D, Fig. S6A). Receptor-ligand pairs, such as SPP1-CD44, CD74-MIF, CD74-COPD, and CD74-APP were considered as strong interaction (Fig. 9E and F, Fig. S6 B and C). Survival analysis indicated that these gene signatures held negative effect on prognosis based on the TCGA-PAAD dataset. The expression level of these gene signatures among different samples indicated that the CD74 associated cell interaction and the TAM function might play important roles in the TME of UCOGCP. In comparison with the rest PDAC samples, CD74 stayed in a higher expression level in multiple cell clusters, indicating the versatile roles of CD74 in UCOGDP development (Fig. S7A). When we knocked-down CD74 on pancreatic cancerous cells in vitro (Fig. S7B), the viability, migration, and invasion of the pancreatic cancerous cells were decreased significantly in comparison with those sh-NC group, indicating an aggressive role of CD74 in pcancreatic cancerous cells (Fig. S7C-H).

## Discussion

Undifferentiated carcinoma with osteoclast-like giant cells of the pancreas (UCOGCP) is a rare type of pancreatic cancer, accounting for less than 1% of total cases diagnosed [1, 25]. UCOGCP tended to be large in size, sometimes reaching 10 cm at the time of diagnosis, along with polypoid growth to the papilla or the main pancreatic duct, or cystic degeneration[1, 17]. Pathology analysis showed that UCOGCPs contain OGCs, and sometimes accompanied with the carcinomatous component. The investigation of the heterogeneity of the TME of UCOGCPs would help tailoring treatment strategy [25].

Accumulating evidence has shown that scRNA-seq is a powerful technology for dissecting the heterogeneity of TME of pancreatic tumors [44, 45]. And scRNA-seq could also determine the histogenesis of special cell clusters, such as osteoclasts in giant cell tumor of bone [46]. In the present work, by machine learning of the gene expression pattern between UCOGCP sample and PDAC samples, the OGCs were found originated from stem-cell-like mesenchymal epithelial cells (SMECs). Functional analysis showed that the OGCs cluster was enriched in antigen presentation, immune response, and stem cell differentiation. Further study revealed the involvement of CD74 in the formation of TAM enriched TME in UCOGCP.

Studies have shown that there are a small number of cells in tissues that can renew themselves, sometimes proliferate and differentiate, and have the characteristics of stem cells [47]. It has been reported that cells expressing cell surface markers CD44, CD24 and EPCAM in pancreatic tumors have the potential to promote the formation, proliferation and metastasis of cancer cells, and these cells were entitled as pancreatic cancer stem cell (CSC) by researchers [41]. It was believed that EMT is associated with the formation of CSCs [48, 49]. In our work, we found no clusters expressed gene markers of osteoclasts in OGCs, such as CTSK, MMP9, OSCAR, and APC5 [46], suggesting that the multinuclear giant cells in UCOGCP were just osteoclast-like cells. From machine leaning, we found distinct clusters originated from ductal special epithelial clusters dominantly belonging to UCOGCP expressing marker genes such as CD68 and KRT81, and the latter were cytokeratin, a cell marker for epithelial cells [50]. IHC staining confirmed that OGCs were CD68 and KRT81 positive, suggesting that OGCs were epithelial origination. Gene marker and GO BP enrichment analysis showed that this cluster of epithelial cells were under active epithelial-mesenchymal transition (EMT), a biological process of epithelial cells with remodeled morphology and improved migration ability [51], which was consistent with previous findings [52]. During EMT, the epithelial elements lose their polarity and cell–cell adhesion, remodeled cell morphology, and acquired migratory capacity. ETM was believed to be associated with undifferentiated carcinomas [53], and signified poor prognosis in PDAC [54]. In this work we found that, the cluster of EMT specially enriched in MYC-TARGET_V1 pathway, and representative gene clusters such as AP3S1, BUB3, EIFD3, LDHA, NMP1, PSMD14, SERBP1, SSBP1, UBE2L3, LOX, SERPINE1, TGFBI, and CD44 that affected the overall survival of PAAD. Further study of these genes and the signaling pathway will assist in better understanding the role of EMT in OGCs formation and potential therapy strategy. Trajectory analysis showed that the OGCs came from EMT clusters expressing CD44, CD24 and EPCAM, which exhibited CSC behavior [41]. Gene marker and GO BP enrichment showed that the OGCs were enriched in antigen presentation, hematopoietic stem cell differentiation and negative regulation of G2/M, indicating that the cell cluster was rather inert and that it might originated from the dysregulation of mitosis. Interestingly, in our work, although the OGCs did not express p53, EPCAM, MUC1, KRT19, they did express certain keratin, such as KRT81, which was not expressed in the EPCAM positive epithelial cluster. Unfortunately, we have not obtained the primary cultures of the OGCs in our work, the complicated interactions between these OGCs and the other cells in the TME need further investigation.

The TME of UCOGCPs were complicated and the investigation of the TME heterogeneity would benefit treatment strategy [25]. Smyth et al. suggested that the TME should be categorized into four types on the number of TILs and the PD-L1 expression level [55]. Luchini et al. found that PD-L1 was more frequently expressed in cases associated with PDAC than in cases associated with pure UC-OGC, and PD-L1-positive UGOGCP was associated with a three-fold higher risk of mortality than PDL1-negative UCOGCP [20]. Recently, PD-L1 expression was confirmed to be highly expressed in UCOGCP and metastatic lung lesions, whereas lymphocytic infiltration was stronger in the lung metastatic lesions than in the primary pancreatic lesion. Moreover, pembrolizumab therapy was effective only in the lung lesion but not in UCOGCP. In contrast, a latest study unveiled that pancreatic UCOGC exhibited a continued response to PD-1/PD-L1 blockade even without resection [56]. Infiltration of CD163 positive tumor-associated macrophages (TAM) played important roles in the formation of the distinct TME in UCOGC [20]. Here, we used scRNA-seq to decrypt TME heterogeneity on UCOGCP and also found the involvement of PD-1 pathway in the UCOGCP (Fig. S8). Moreover, we found that UCOGC was affluent in tumor associated myeloid cells, endothelial cells, and fibroblasts. Distinct cell clusters could be identified in these types of cells based on machine learning, which were involved in angiogenesis, immune responses, and leucocyte migration. These cells together form a feedback circle to strengthen the unique tumor microenvironment. Interestingly, marker genes highly expressed in the UCOGC clusters exert undistinguished effects on tumor prognosis at the early stage, however, the overall survival rate dropped sharply with the progression of the tumor, which might explain the controversial results regarding the controversial prognosis of the UCOGC compared with PDAC [17]. CD74 was considered as an MHC class II chaperone, functioning as an antigen presentation server. Besides, CD74 also participated in endosomal trafficking, cell migration and cellular signaling through interacting with the macrophage migration inhibitory factor (MIF) [57]. Moreover, CD74 could recruit CD44 or CXCR4, and such complex could trigger subsequent events [57]. Upregulation of CD74 was reported to regulate the progression and maintenance of gastrointestinal neoplasia [58]. Besides, CD74 was associated with perineural invasion (PNI) and poor survival of patients with PDAC [59, 60]. In this work, we found strong interaction between the EMT epithelial cell clusters and other cell clusters, especially the recruitment of TAM. Moreover, the expression of CD74 in UCOGC was significantly higher than the rest of PDAC samples, the high level expression of CD74 in pancreatic cancerous cells held aggressive effects, indicating that it could be a decent therapeutic target for UCOGC treatment.

Our research suggested the histogenesis and the function of the OCGs in UCOGCP, the TAM enriched characteristics of the TEM, as well as the potential roles of CD74 in this special tumor microenvironment. However, due to the rarity of the case type and the requirements of samples for scRNA-seq [61], we only successfully examined one UCOGCP sample in the current work. Therefore, more UCOGCP samples examined by scRNA-seq and/or IHC staining should be carried out in the future to verify our finding.

## Conclusions

Overall, our study characterizes the heterogeneity of UCOGC, providing evidence of the genesis of the OGCs. By unveiling the single-cell transcription profile of UCOGC, we reveal CD74 could be a novel clinical candidate for the diagnosis, prognosis, and treatment of UCOGC.

## Supplementary Information

**Additional file 1: Fig. S1.** Clustering and annotation of the scRNA-seqdata of samples from undifferentiated carcinoma with osteoclast-like pancreaticgiant cells (UCOGCP) and other pancreatic ductal adenocarcinoma (PDAC). A, PCA analysis of scRNA-seq data from different samples before batchnormalization (BN). B, UMAP reduction denoted the elimination of batch effectsamong scRNA-seq data from different samples. Harmony R package were used forBN. C, Cell cycle estimation. D, Flow chart described the present work. UCOGCPand PDAC samples are dissociated into single cells, captured in 10× genomicplatform for library construction and RNA sequencing. The sequencing resultswere then undergoing bioinformatics analysis after QC, normalization, PCA. B,Uniform manifold approximation and projection (UMAP) showing major clusterslearned in Seurat package (4.0.4) in R (4.0.5). D, Top three markers of eachcluster obtained from “FindAllMarkers” function fromSeurat package (4.0.4) were shown in dop plot. E and F, Classic cell annotationmarkers were shown in violin plot and dim plot, respectively. G, Thecomposition of clusters identified. H, The distribution of each cluster from differentsamples. I, Cluster tree of the nineteen clusters with distinct gene expressionpatterns at the resolution of 0.6.

**Additional file 2: Fig. S2.** The role of LOX, SPERINE1, CD44, and TGFBI inpancreatic cancer cell line PANC-1 and SW1990. A,The expression pattern of the gene markers in different pancreatic cell lines.B and C, The expression level of gene markers after knocked-down. D and E, CCK8analysis of cells knocked-down these gene markers. F-H, Apoptosis analysis.I-K, Cell colony formation assay. L-Q,Transwell assay.

**Additional file 3: Fig. S3.** Clustering and annotation of the scRNA-seqdata of UCOGCP sample (pca_ai1). A,Clustering tree of pca_ai1 mate data under different resolutions. B, UMAPshowing major clusters of pca_ai1 learned in Seurat package (4.0.4) in R(4.0.5). C, Top three markers of each cluster obtained from “FindAllMarkers”function from Seurat package (4.0.4) were shown in dop plot. D, Cell cycleestimation. E, The annotations enriched in BaronPancreasData dataset usingSingle R package. F, Classic cell annotation markers were shown in violin. Gand H, Mapping of cluster 15 in Fig.2. I, Cell markers in cluster 15 in Fig. 2were shown in violin plot.

**Additional file 4: Fig. S4.** Mapping of the interested clusters in theepithelial cell re-clustering UMAP of the UCOGCP sample (pca_ai1). A and B, Mapping of the ductal clusters in Fig. 2 to the epithelialcell reclustering UMAP of the UCOGCP sample (pca_ai1). C and D, Mapping of theductal clusters in Fig. 3 to the epithelial cell reclustering UMAP of theUCOGCP sample (pca_ai1).

**Additional file 5: Fig. S5.** Mapping of theinterested clusters from trajectory analysis in the epithelial cell re-clusteringUMAP of the UCOGCP sample (pca_ai1). A,The states of the trajectory analysis under DDRTree reduction. B, The clustersof the scRNA-seq metadata from UCOGCP sample (pca_ai1) learned by Seuratpackage (4.0.4) in R (4.0.5) under the resolution of 0.8. C-E, Mapping of thestates learned by trajectory analysis. in B.

**Additional file 6: Fig. S6.** Cell communication analysis of UCOGCP sample(pca_ai1). A, Major ligand–receptor interaction amonginterested cell clusters. B and C Ligand–receptor pairs shown in a bubble plot.

**Additional file 7: Fig. S7.** The role of CD74 in pancreatic cancer cellline PANC-1. A, The expression pattern of CD74 in differentcell types in all samples. B, The expression level of CD74 after knocked-down.C, CCK8 analysis of cells knocked-down CD74. D-F, Transwell assay of cellsknocked-down CD74. G and H, Wound healing assay of cells knocked-down CD74.

**Additional file 8: Fig. S8.** Heterogeneity of lymphocytes cells. A, Major clusters of the tumor associated fibroblast cells of allsamples were shown in UMAP. B, Top three markers of each cluster obtained from“FindAllMarkers” function from Seurat package (4.0.4) were shown in dop plot.C-E, The distribution of each cluster in each sample were shown in balloonplot, heatmap, and UMAP, respectively. F, Wikipathway enrichment among clusterswere shown in dot plot. G, Survival analysis of gene signatures in IL-18signaling pathway in cluster 1 in pancreatic cancer using TCGA-PAAD on websiteGepia2 (http://gepia2.cancer-pku.cn/#index).

**Additional file 9: Table S1.** Clinical data of the samples in this work.

**Additional file 10: Table S2.** DEGs of clusters at the resolution of 0.1.

**Additional file 11: Table S3.** DEGs of clusters at the resolution of 0.6.

**Additional file 12: Table S4.** DEGs of clusters of ductal type I subclusters at the resolution of 0.1.

**Additional file 13: Table S5.** Hallmarks of ductal type I recluster at resolution of 0.1.

**Additional file 14: Table S6.** gesGO analysis of cluster3 in figure 3.

**Additional file 15: Table S7.** DEGs of clusters 3 in figure 3 at the resolution of 0.1.

**Additional file 16: Table S8.** GO BP analysis of clusters in figure 4.

**Additional file 17: Table S9.** GO BP analysis of gene modules of clusters 3 in figure 4.

**Additional file 18: Table S10.** DEGs of pca_ai1 ductal cell clusters.

**Additional file 19: Table S11.** beam_markers.

**Additional file 20: Table S12.** GO BP analysis of beam markers.

**Additional file 21: Table S13.** DEGs of myeloid cell clusters.

**Additional file 22: Table S14.** WIKIpathway analysis of clusters in myeloid cell clusters.

**Additional file 23: Table S15.** 

**Additional file 24: Table S16.** GO BP analysis of clusters of fibroblasts.

**Additional file 25: Table S17.** DEGs of endothelial cell clusters with the resolution of 0.1.

**Additional file 26: Table S18.** DEGs of clusters in pca_ai.

## Data Availability

The datasets used and/or analysed during the current study are available from the corresponding author on reasonable request.

## Abbreviations

OGCs: Osteoclast-like Giant Cells
UCOGC: Undifferentiated Carcinoma With Osteoclast-like Giant-Cells
UCOGCP: Undifferentiated Carcinoma With Osteoclast-like Giant Cells of Pancreas
PDAC: Pancreatic Ductal Adenocarcinoma
scRNA-seq: Single-cell RNA sequencing
IHC: Immunohistochemical
SMECs: Stem-cell-like Mesenchymal Epithelial Cells
TME: Tumor Microenvironment
MCN: Mucinous Cystic Tumors
MCHs: Mononuclear Histiocytes
TAM2: Tumor-Associated Macrophages
GEMs: Gel bead-in-emulsions
UMAP: Uniform Manifold Approximation and Projection
TFs: Transcription Factors
