# Supplementary figures and images for: Single-cell RNA-seq reveals the genesis and heterogeneity of tumor microenvironment in pancreatic undifferentiated carcinoma with osteoclast-like giant-cells
Source: Mol Cancer. 2022 Jun 22;21:133. doi: 10.1186/s12943-022-01596-8 (PMC9214989; doi:10.1186/s12943-022-01596-8)

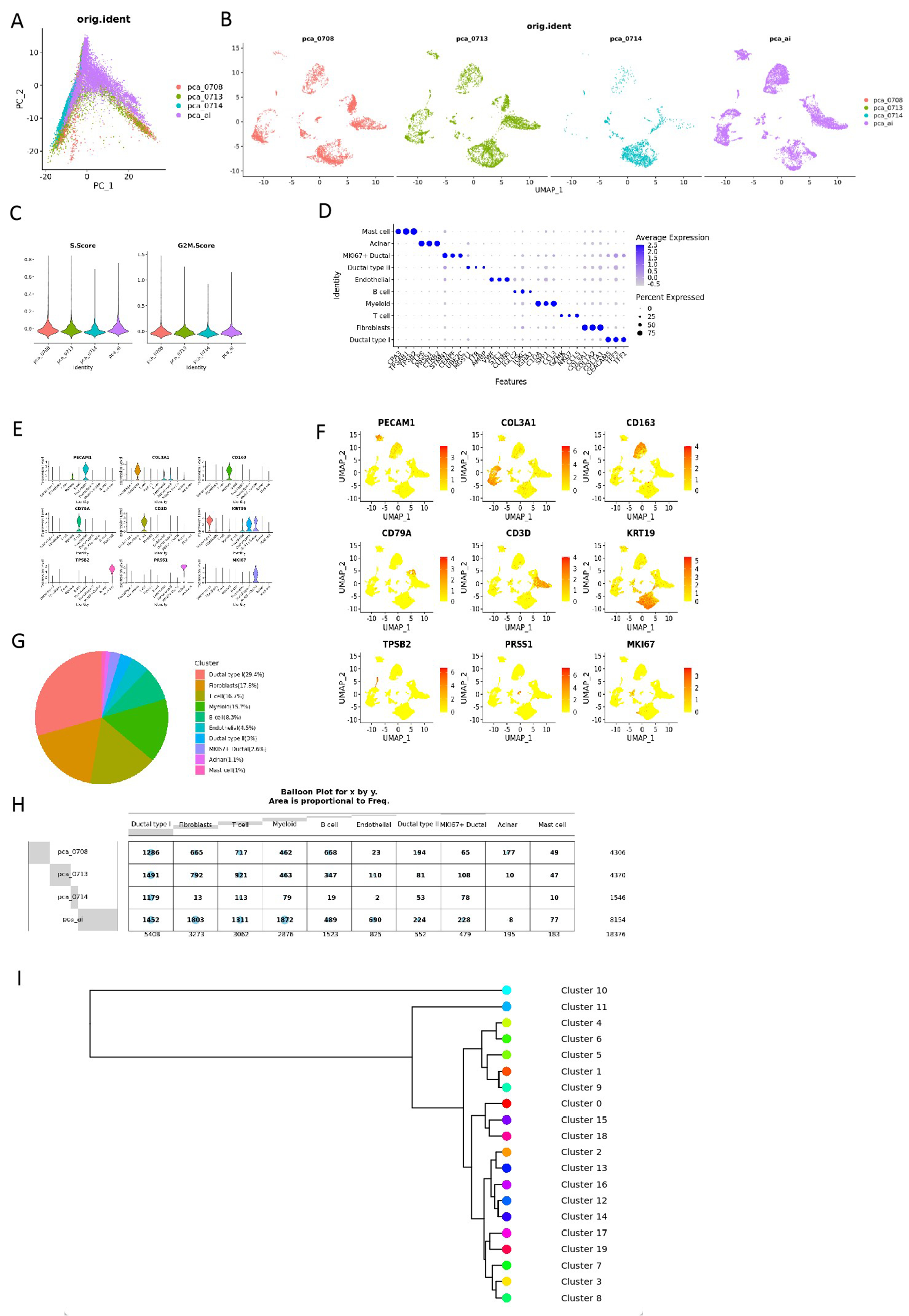

Supplement: Supplementary file 1 — Additional file 1: Fig. S1. Clustering and annotation of the scRNA-seqdata of samples from undifferentiated carcinoma with osteoclast-like pancreaticgiant cells (UCOGCP) and other pancreatic ductal adenocarcinoma (PDAC). A, PCA analysis of scRNA-seq data from different samples before batchnormalization (BN). B, UMAP reduction denoted the elimination of batch effectsamong scRNA-seq data from different samples. Harmony R package were used forBN. C, Cell cycle estimation. D, Flow chart described the present work. UCOGCPand PDAC samples are dissociated into single cells, captured in 10× genomicplatform for library construction and RNA sequencing. The sequencing resultswere then undergoing bioinformatics analysis after QC, normalization, PCA. B,Uniform manifold approximation and projection (UMAP) showing major clusterslearned in Seurat package (4.0.4) in R (4.0.5). D, Top three markers of eachcluster obtained from “FindAllMarkers” function fromSeurat package (4.0.4) were shown in dop plot. E and F, Classic cell annotationmarkers were shown in violin plot and dim plot, respectively. G, Thecomposition of clusters identified. H, The distribution of each cluster from differentsamples. I, Cluster tree of the nineteen clusters with distinct gene expressionpatterns at the resolution of 0.6. [file 12943_2022_1596_MOESM1_ESM.jpg]

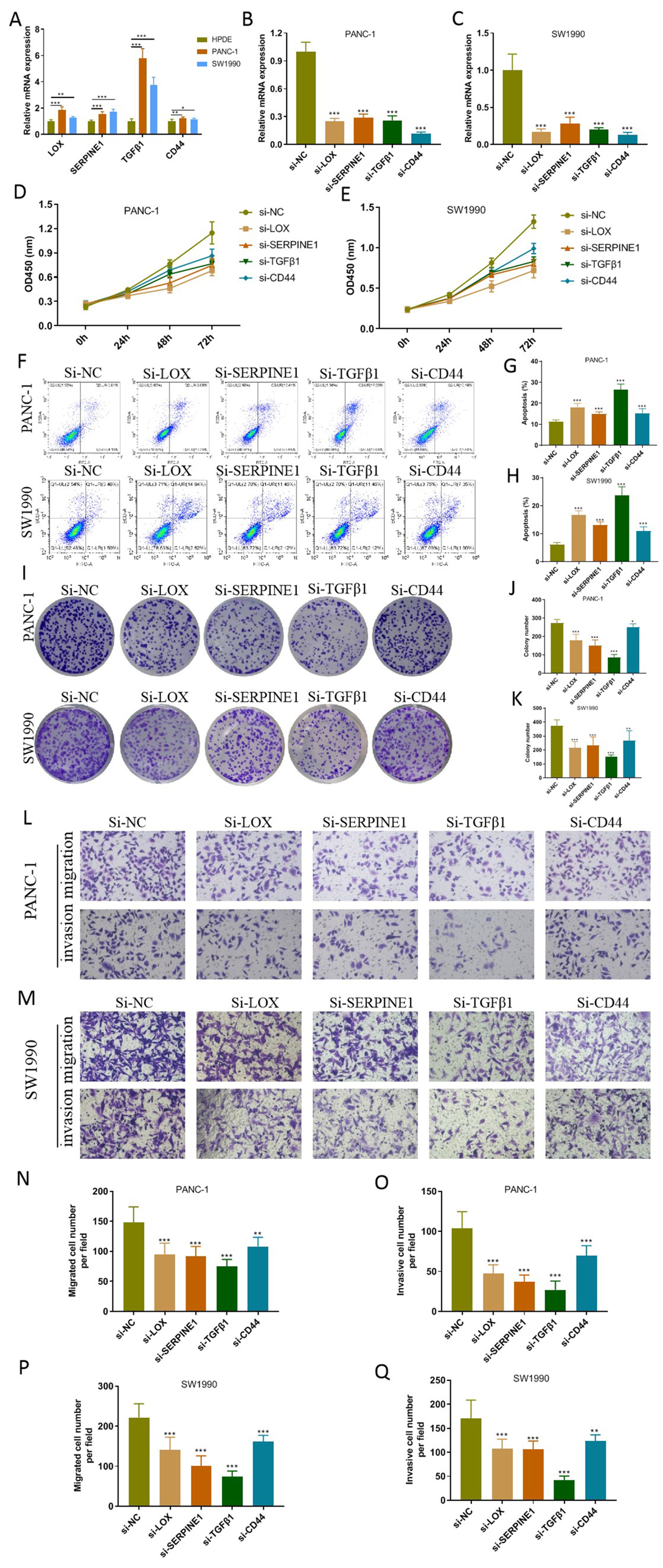

Supplement: Supplementary file 2 — Additional file 2: Fig. S2. The role of LOX, SPERINE1, CD44, and TGFBI inpancreatic cancer cell line PANC-1 and SW1990. A,The expression pattern of the gene markers in different pancreatic cell lines.B and C, The expression level of gene markers after knocked-down. D and E, CCK8analysis of cells knocked-down these gene markers. F-H, Apoptosis analysis.I-K, Cell colony formation assay. L-Q,Transwell assay. [file 12943_2022_1596_MOESM2_ESM.jpg]

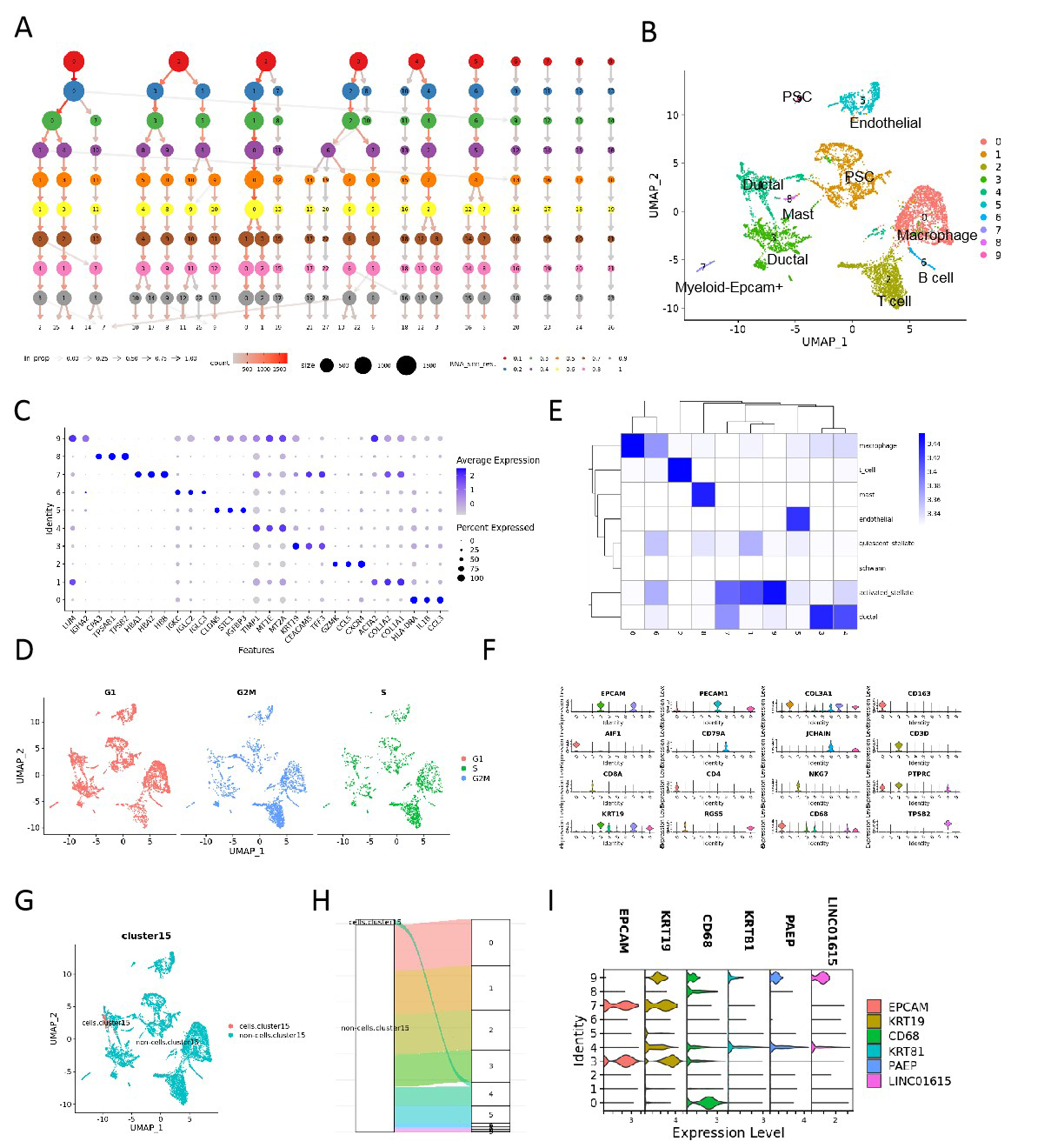

Supplement: Supplementary file 3 — Additional file 3: Fig. S3. Clustering and annotation of the scRNA-seqdata of UCOGCP sample (pca_ai1). A,Clustering tree of pca_ai1 mate data under different resolutions. B, UMAPshowing major clusters of pca_ai1 learned in Seurat package (4.0.4) in R(4.0.5). C, Top three markers of each cluster obtained from “FindAllMarkers”function from Seurat package (4.0.4) were shown in dop plot. D, Cell cycleestimation. E, The annotations enriched in BaronPancreasData dataset usingSingle R package. F, Classic cell annotation markers were shown in violin. Gand H, Mapping of cluster 15 in Fig.2. I, Cell markers in cluster 15 in Fig. 2were shown in violin plot. [file 12943_2022_1596_MOESM3_ESM.jpg]

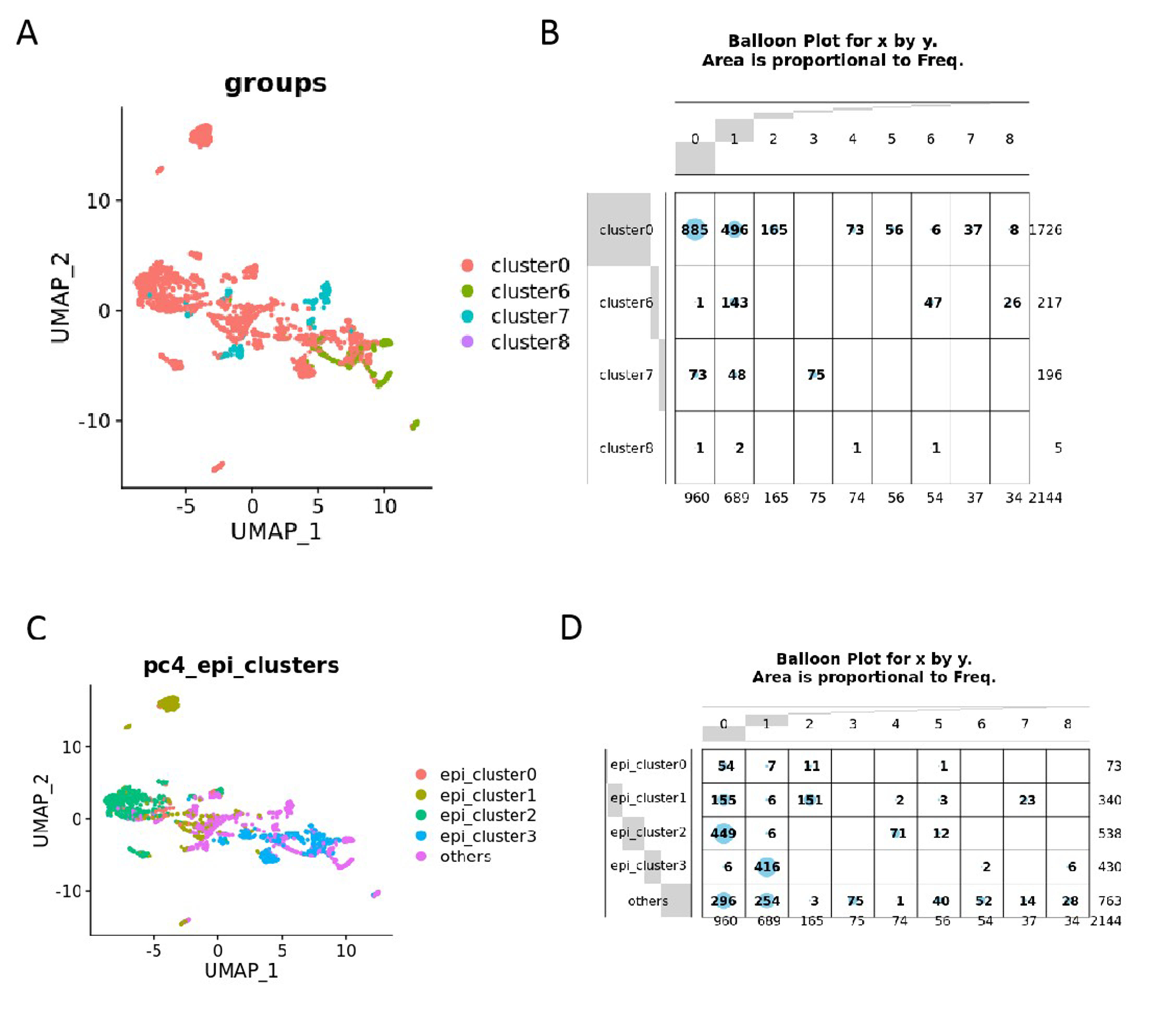

Supplement: Supplementary file 4 — Additional file 4: Fig. S4. Mapping of the interested clusters in theepithelial cell re-clustering UMAP of the UCOGCP sample (pca_ai1). A and B, Mapping of the ductal clusters in Fig. 2 to the epithelialcell reclustering UMAP of the UCOGCP sample (pca_ai1). C and D, Mapping of theductal clusters in Fig. 3 to the epithelial cell reclustering UMAP of theUCOGCP sample (pca_ai1). [file 12943_2022_1596_MOESM4_ESM.jpg]

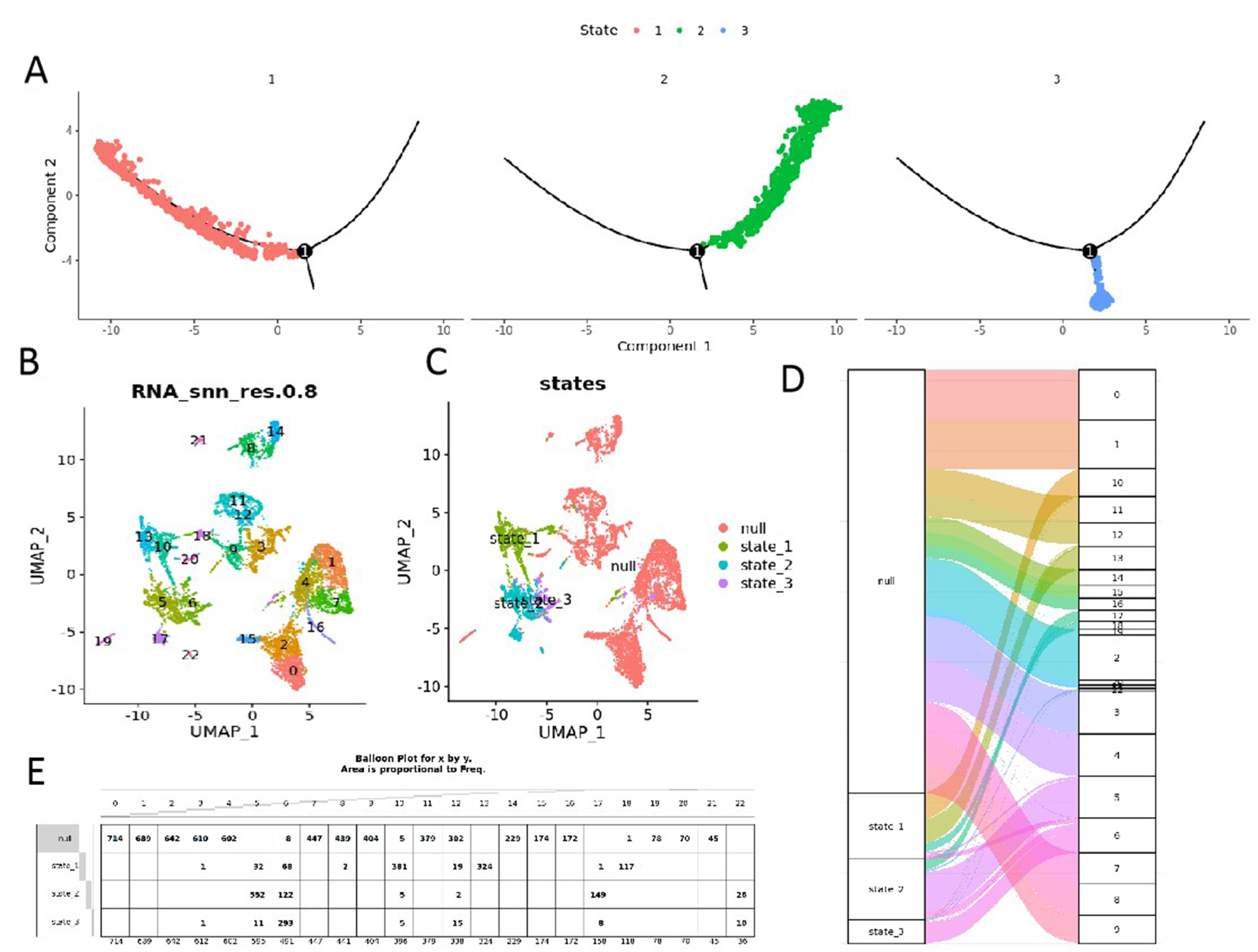

Supplement: Supplementary file 5 — Additional file 5: Fig. S5. Mapping of theinterested clusters from trajectory analysis in the epithelial cell re-clusteringUMAP of the UCOGCP sample (pca_ai1). A,The states of the trajectory analysis under DDRTree reduction. B, The clustersof the scRNA-seq metadata from UCOGCP sample (pca_ai1) learned by Seuratpackage (4.0.4) in R (4.0.5) under the resolution of 0.8. C-E, Mapping of thestates learned by trajectory analysis. in B. [file 12943_2022_1596_MOESM5_ESM.jpg]

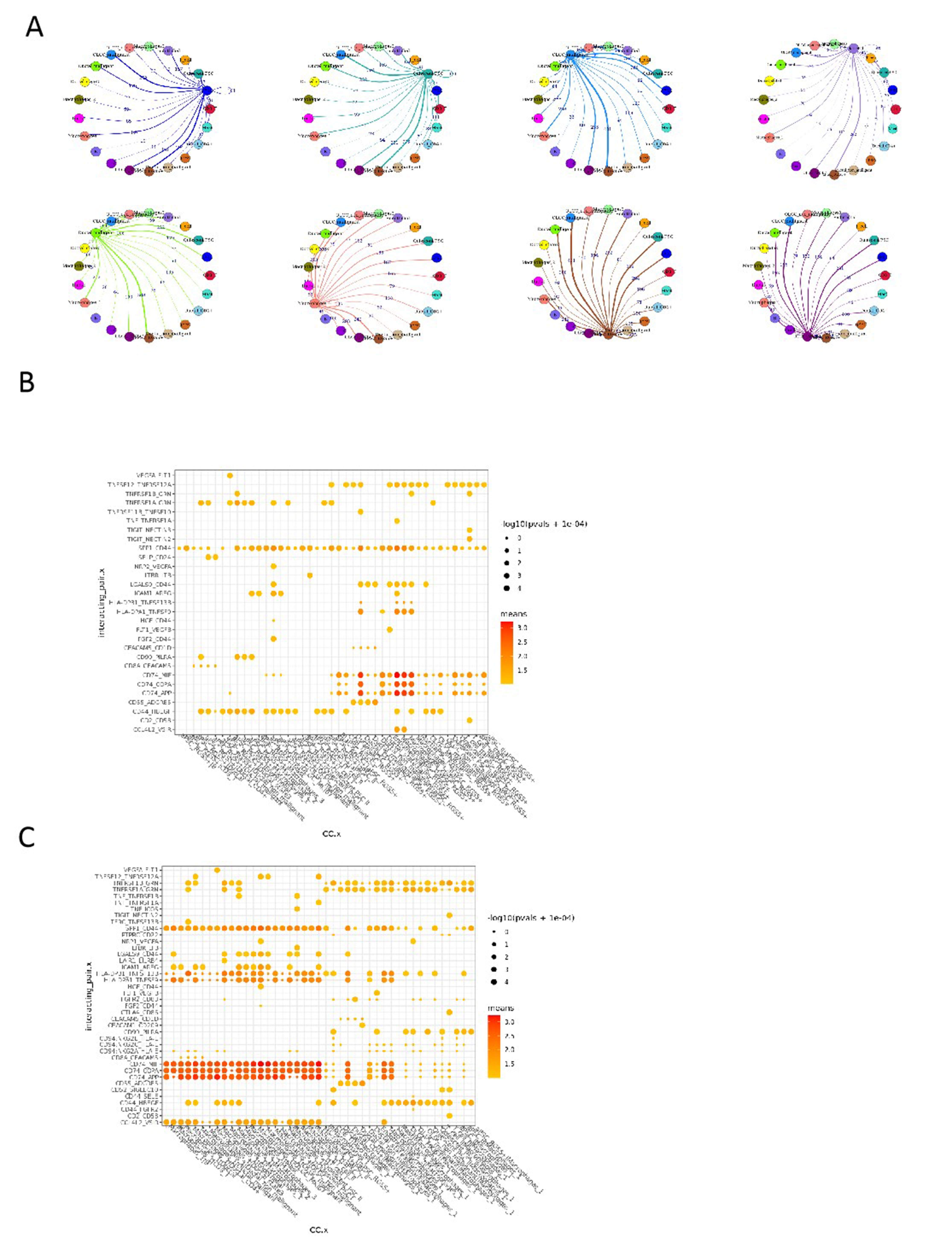

Supplement: Supplementary file 6 — Additional file 6: Fig. S6. Cell communication analysis of UCOGCP sample(pca_ai1). A, Major ligand–receptor interaction amonginterested cell clusters. B and C Ligand–receptor pairs shown in a bubble plot. [file 12943_2022_1596_MOESM6_ESM.jpg]

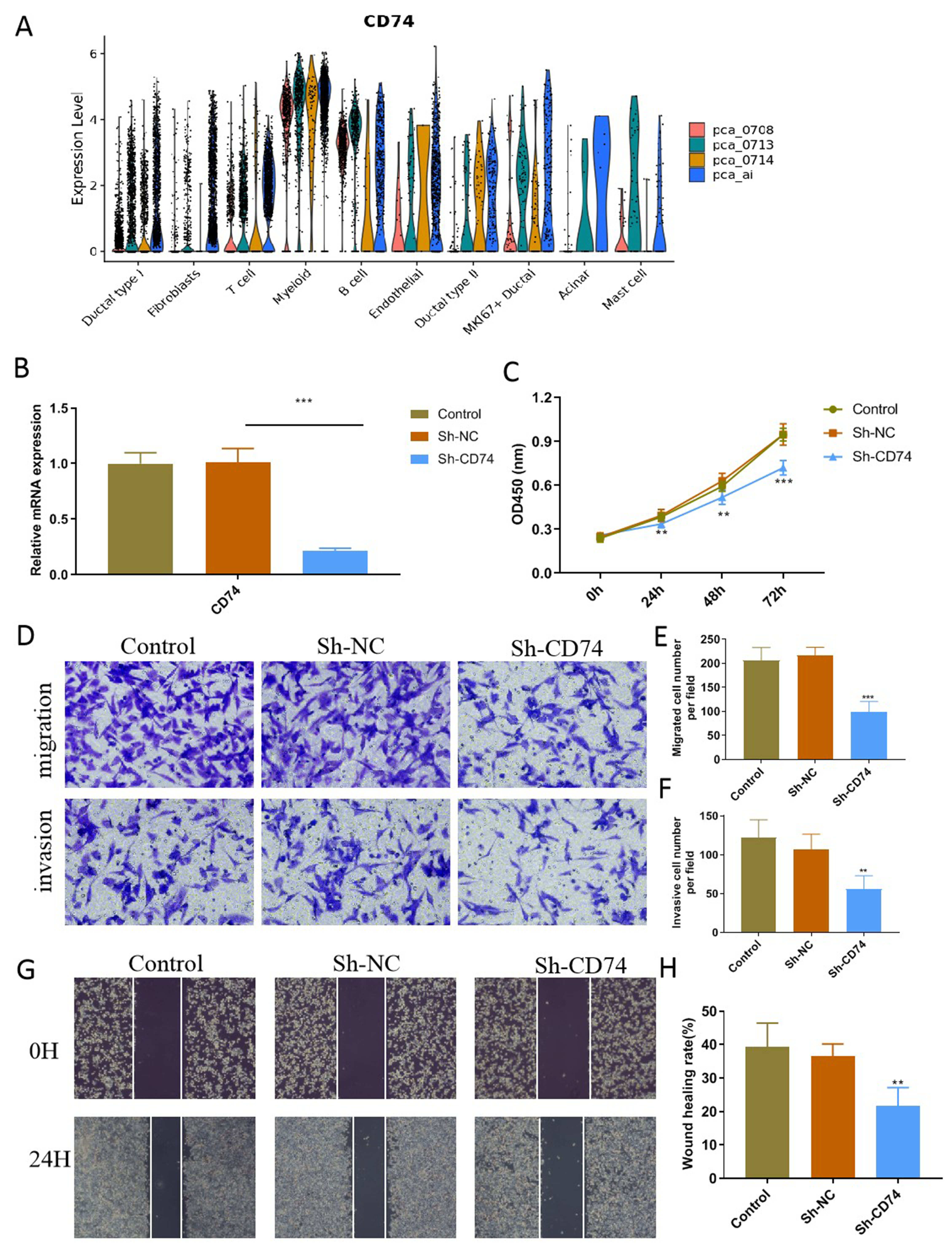

Supplement: Supplementary file 7 — Additional file 7: Fig. S7. The role of CD74 in pancreatic cancer cellline PANC-1. A, The expression pattern of CD74 in differentcell types in all samples. B, The expression level of CD74 after knocked-down.C, CCK8 analysis of cells knocked-down CD74. D-F, Transwell assay of cellsknocked-down CD74. G and H, Wound healing assay of cells knocked-down CD74. [file 12943_2022_1596_MOESM7_ESM.jpg]

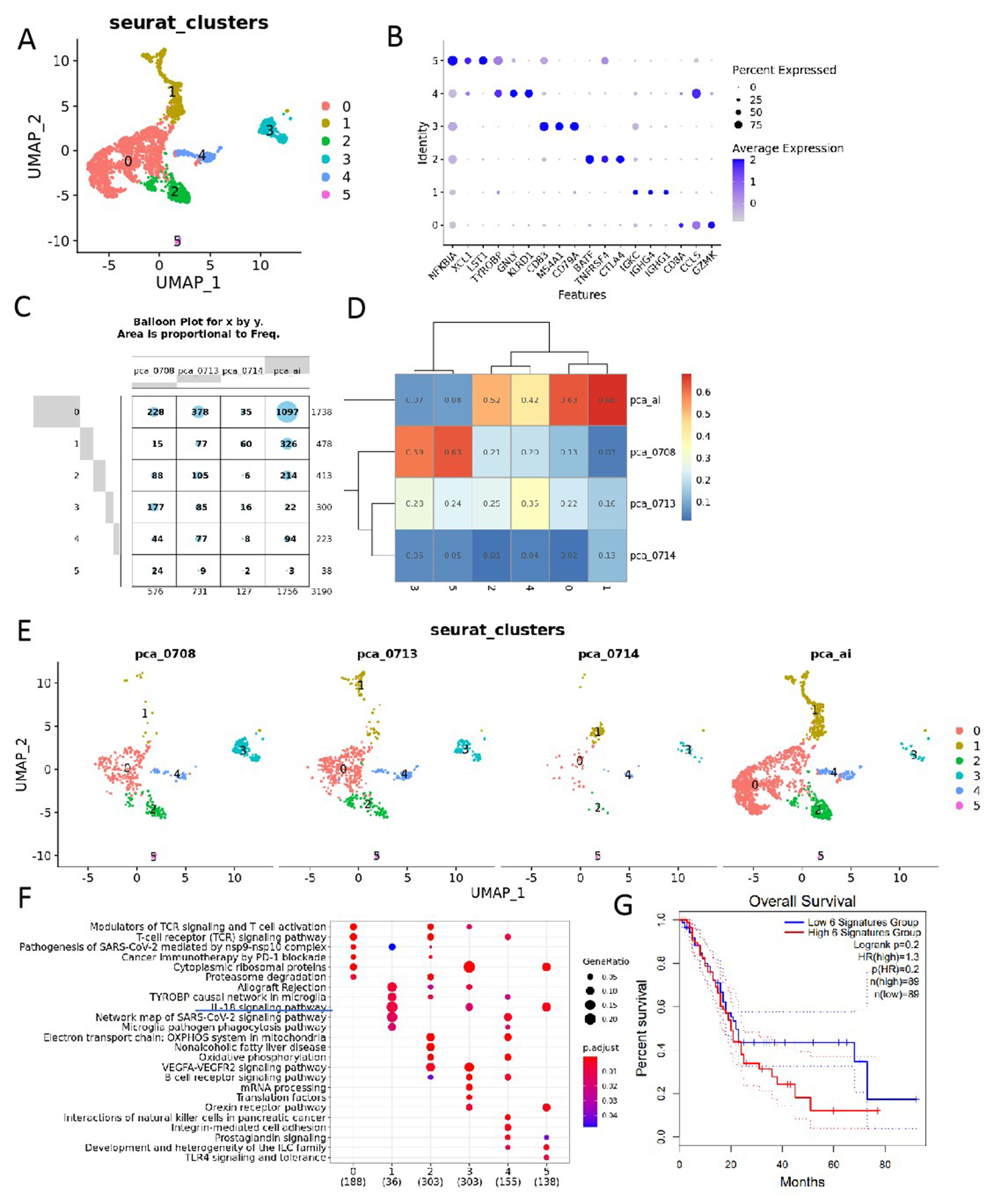

Supplement: Supplementary file 8 — Additional file 8: Fig. S8. Heterogeneity of lymphocytes cells. A, Major clusters of the tumor associated fibroblast cells of allsamples were shown in UMAP. B, Top three markers of each cluster obtained from“FindAllMarkers” function from Seurat package (4.0.4) were shown in dop plot.C-E, The distribution of each cluster in each sample were shown in balloonplot, heatmap, and UMAP, respectively. F, Wikipathway enrichment among clusterswere shown in dot plot. G, Survival analysis of gene signatures in IL-18signaling pathway in cluster 1 in pancreatic cancer using TCGA-PAAD on websiteGepia2 (http://gepia2.cancer-pku.cn/#index). [file 12943_2022_1596_MOESM8_ESM.jpg]
