# Supplementary material for: Single-cell RNA-seq reveals the genesis and heterogeneity of tumor microenvironment in pancreatic undifferentiated carcinoma with osteoclast-like giant-cells
Source: Mol Cancer. 2022 Jun 22;21:133. doi: 10.1186/s12943-022-01596-8 (PMC9214989; doi:10.1186/s12943-022-01596-8)
Supplement: Supplementary file 9 — Additional file 9: Table S1. Clinical data of the samples in this work. [file 12943_2022_1596_MOESM9_ESM.docx]

**Table S1 Clinical data of the samples in this work.**

| ID | Gender | Age | Pathological feature |
| --- | --- | --- | --- |
| Pca_0708 | F | 68 | PDAC |
| Pca_0713 | M | 58 | PDAC |
| Pca_0714 | F | 55 | PDAC |
| Pca_ai | F | 59 | Mucinous adenocarcinoma with UCOGC |
